# Human Stem Cell-Derived TRPV1-Positive Sensory Neurons: A New Tool to Study Mechanisms of Sensitization

**DOI:** 10.3390/cells11182905

**Published:** 2022-09-17

**Authors:** Katrin Schrenk-Siemens, Jörg Pohle, Charlotte Rostock, Muad Abd El Hay, Ruby M. Lam, Marcin Szczot, Shiying Lu, Alexander T. Chesler, Jan Siemens

**Affiliations:** 1 Department of Pharmacology, Im Neuenheimer Feld 366, University of Heidelberg, 69120 Heidelberg, Germany; 2 Department of Translational Disease Understanding, Grünenthal GmbH, Zieglerstr. 6, 52078 Aachen, Germany; 3 Ernst Strüngmann Institute, Deutschordenstr. 46, 60528 Frankfurt, Germany; 4 National Center for Complementary and Integrative Health, NIH, 35A Convent Drive, Bethesda, MD 20892, USA; 5 Center for Social and Affective Neuroscience, Department of Clinical and Experimental Medicine, Linköping University, 58330 Linköping, Sweden; 6 Oliver Wyman GmbH, Muellerstr. 3, 80469 Munich, Germany

**Keywords:** somatosensation, human pluripotent stem cells, nociceptor-like cells, homogenous neuronal population, TRPV1 responders, translational tool

## Abstract

Somatosensation, the detection and transduction of external and internal stimuli such as temperature or mechanical force, is vital to sustaining our bodily integrity. But still, some of the mechanisms of distinct stimuli detection and transduction are not entirely understood, especially when noxious perception turns into chronic pain. Over the past decade major progress has increased our understanding in areas such as mechanotransduction or sensory neuron classification. However, it is in particular the access to human pluripotent stem cells and the possibility of generating and studying human sensory neurons that has enriched the somatosensory research field. Based on our previous work, we describe here the generation of human stem cell-derived nociceptor-like cells. We show that by varying the differentiation strategy, we can produce different nociceptive subpopulations with different responsiveness to nociceptive stimuli such as capsaicin. Functional as well as deep sequencing analysis demonstrated that one protocol in particular allowed the generation of a mechano-nociceptive sensory neuron population, homogeneously expressing TRPV1. Accordingly, we find the cells to homogenously respond to capsaicin, to become sensitized upon inflammatory stimuli, and to respond to temperature stimulation. The efficient and homogenous generation of these neurons make them an ideal translational tool to study mechanisms of sensitization, also in the context of chronic pain.

## 1. Introduction

Our ability to detect and transduce stimuli from the external and internal environment drives a major part of our daily behavior. The perception of innocuous mechanical stimuli for example, enables us to discriminate various textures and vibrations. We can perceive a friendly touch on our arm as comforting and the warmth of the sun on our bare skin as pleasant. At the same time, we can perceive extreme temperatures, chemical compounds or mechanical forces as painful, leading to protective reflexive withdrawal responses to avoid tissue damage [1]. The cells responsible for the detection and transduction of the respective stimuli are sensory neurons located in the dorsal root or trigeminal ganglia (DRG & TG). The plethora of stimuli that we are able to detect is reflected in the diversity of sensory neuron subtypes we have. Nociception, the ability to detect and transduce painful stimuli, is of special interest, as the primary function of pain as a warning system can outlive its usefulness by becoming chronic. According to some studies, up to 30% of the worldwide population are suffering from chronic pain which is a burden for the affected person as well as the health system [2]. Therefore, the need to understand the underlying mechanisms why pain becomes chronic in certain patients is unbowed.

Primary human sensory neurons are difficult to access and therefore did not have a major role as a model system to study human nociception. This has changed tremendously with the accessibility of human embryonic and induced pluripotent stem cells (hESCs & hiPSCs), which now offer the unprecedented opportunity to derive sensory neurons from pain-afflicted patients. Certainly, this depends on suitable, efficient procedures to differentiate the cells. A few protocols have been published, describing how to generate sensory neurons pursuing different strategies: either small molecule inhibitors are used to drive stem cells towards sensory neuron fate [3] or overexpression of one or more transcription factors, directly reprogramming human fibroblasts or stem cells to become sensory neurons [4,5] or a combination thereof [6].

We have chosen a different path, by generating the progenitor cells of sensory neurons—neural crest cells—as starting material for subsequent differentiation by viral expression of *NEUROGENIN1* (*NGN1*). NGN1 is a basic helix loop helix factor critical for nociceptor development in rodents [7]. The differentiation process was further modulated by the addition of morphogens and neurotrophic factors. NGN1 expression had already been used before, either in combination with other transcription factors, or alone in order to generate peripheral neuron-like cells from human pluripotent stem cells or fibroblasts [4,5,6].

Native nociceptors are not a homogenous cell population; rather, they are molecularly diverse, reflecting their ability to respond to different types of stimuli and differentially mediate and modulate painful responses [8]. Within our differentiation paradigm, we diversified the generated nociceptors by varying the duration of *NGN1* expression and by exposing the differentiating cells to different extracellular factors.

The thereby generated neurons showed molecular and functional characteristics of nociceptors. One differentiation protocol in particular generated a homogenous population of sensory neurons expressing TRPV1, a prototypical molecular marker of nociceptors and a major driver of inflammatory pain [9]. Accordingly, we find the cells to homogenously respond to capsaicin and to have the potential for becoming sensitized upon inflammatory stimulation. Both of these characteristics are hallmarks of native nociceptors and a notorious feature that has been reported to drive/fuel pathological forms of pain [8,10]. Additionally, these neurons respond to temperature stimulation, in agreement with the expression of multiple thermosensitive TRP ion channels, including TRPV1.

Similar to the abundance of mechanosensitive nociceptors in DRG cultures of mouse many of the derived cells were inherently mechanosensitive. Furthermore, many of the differentiated neurons expressed PIEZO2, a protein recently identified as a key driver of mechanotransduction in low-threshold mechanoreceptors in mice and humans [11,12,13]. Deletion of the *PIEZO2* gene abolished mechanotransduction in the hESC-derived nociceptors. This was a finding that could be corroborated in analogue experiments using cultured mouse nociceptors devoid of PIEZO2, suggesting that this receptor might also be involved in mechanotransduction in a selective subset of mouse and human nociceptive neurons in vitro.

In summary, we here describe the comparison of several procedures for the generation of diverse populations of human nociceptor-like cells, with one protocol in particular resulting in the production of a homogenous population of TRPV1-expressing neurons, showing molecular and functional characteristic hallmarks of pain-sensing neurons. The efficient and reliable generation of this specific neuronal subtype makes them an ideal tool to study aspects of sensitization in a human cell-based context that could help to better understand the molecular and functional changes in nociceptive function that might result in pathological states of sensitization.

## 2. Materials and Methods

### 2.1. Generation of hESC-Derived Sensory Neurons

#### 2.1.1. Generation of hESC-Derived Neural Crest-like Cells (NCLCs; Protocol Adapted from [14])

Use of human embryonic stem cells (HUES-7 line; Harvard University, Cambridge, MA, USA) was approved by the German Central Ethics Committee for Stem Cell Research (project codes: #1710-79-1-4-51 and 1710-79-1-4-51-E01). A quantity of hESCs were cultured on Matrigel (Corning)-coated dishes in E8-medium (self-made according to Chen et al., 2011) at 37 °C and 5% CO_2_ until 80% confluency. Cells were detached using EDTA (0.5 mM) and transferred onto uncoated dishes in sphere medium (DMEM:F12, Neurobasal, N2-supplement, B27-supplement, glutamine (all ThermoFisher Scientific, Waltham, MA, USA)), insulin (CSBio) with freshly added EGF and FGF (20 ng/μL; Peprotech). ROCK inhibitor (10 μM; Stem Cell Technologies) was added for up to 48 h, depending on size of spheres. One day after plating, small spheres become visible. The medium was changed every other day. After 7–9 days, spheres spontaneously attach and NCLCs start to migrate out. NCLCs were collected using Accutase (Sigma) after remnants of spheres were manually removed from the plate. NCLCs can be frozen in liquid nitrogen or immediately plated on Poly-D-ornithine (15 μg/μL, Sigma), laminin (10 μg/mL, Sigma) and fibronectin (10 μg/mL, ThermoFisher Scientific)-coated 3.5 cm dishes in a density of 60,000 cells/cm^2^ in sphere medium without FGF and EGF.

#### 2.1.2. Generation of Low-Threshold Mechanoreceptors (LTMRs)

LTMRs were generated as described by Schrenk–Siemens et al., 2015 [12]. In brief: NCLCs were cultured in a differentiation medium (sphere medium supplemented with BDNF, GDNF, NGF and NT3 (all 10 ng/μL, Peprotech) and retinoic acid (0.1 μM, Sigma). The medium was changed daily and every second day starting after two weeks. LTMRs were used for experiments after at least 21 days in culture.

#### 2.1.3. Generation of Nociceptor-like Cells (NOCL1-3) Using Induced NGN1 Expression

To generated NOCL1-3, NCLCs were infected with two lentiviruses: one, expressing rtTA under the control of a ubiquitin promoter for constitutive expression of rtTA. The other, expressing *NGN1*, *EGFP*, and a puromycin resistance cassette as a fusion protein, linked via P2A and T2A sequences, under the control of TeTO promoter. Cells were infected in sphere medium, containing HEPES (10 mM, ThermoFisher Scientific, Waltham, MA, USA) and protamine sulphate (8 μg/mL). After 6 h, cells were washed three times with PBS and kept in a sphere medium until the next day. The next day, the medium was changed to differentiation medium (sphere medium containing BDNF, NGF and GDNF (10 ng/mL; Peprotech), Antibiotic-Antimycotic (1x; ThermoFisher Scientific) and in the case of NOCL1 and NOCL2 also 5% fetal calf serum (ThermoFisher Scientific). *NGN1* expression was induced by addition of doxycycline (2 μg/mL, Sigma, St. Louis, MO, USA) for 10 days (NOCL1 and 3) or for the whole culture period (NOCL2). On the second day of induction, puromycin (10 μg/mL, Invivogen) was added (NOCL3) for 24–48 h to eliminate those cells that were not infected by the virus. After 4 to 5 days of induction, cells were detached using Accutase (Sigma) and plated on freshly laminin/fibronectin-coated dishes or glass coverslips in a density of 31,000 cells/cm^2^. After 13 days in culture, cells were treated with Mitomycin C (10 μg/mL, Sigma) for 45 min, to prevent proliferation of any non-neuronal cells still in the culture, then washed carefully twice with PBS and kept in a differentiation medium for at least 21 days in total. The medium was changed every day for the first 10 days and then only every other day.

#### 2.1.4. Generation of Nociceptor-like Cells Using NGN1, ISL2 and KLF7 (NOCL4)

Modified from the protocol published by Wainger et al., 2015 [5], NOCL4 cells were generated by infecting NCLCs with three lentiviruses, expressing constitutively *NGN1*, *ISL2* and *KLF7*. Cells were infected for 6 h, then washed with PBS and kept in a sphere medium until the next day. The medium was then changed to a differentiation medium (as above), including 5% fetal calf serum (ThermoFisher Scientific). Neurons were kept in culture for at least 21 days before experiments were done.

### 2.2. Virus Generation

Lentiviruses were produced in HEK293TN cells (Biocat) by cotransfection with three helper plasmids (pRSV-REV, pMDLg/pRRE and pMD2.G, obtained from Dr. Alexander Löwer, Berlin, Germany) plus the lentiviral vector DNA: FUW-TetO-Ngn1-P2A-EGFP-T2A-Puro, FUW-rtTA (obtained and modified from Dr. Thomas Südhof, Stanford University, Palo Alto, CA, USA); pMXs-dest-WRE-Ngn1, pMXs-dest-WRE-Klf7, pMXs-dest-WRE-Isl2 (obtained from Dr. Clifford Woolf, Harvard Medical School, Boston, MA, USA); ELFa-Runx1-IRES-GFP and ELFa-TRKA-IRES-GFP using calcium phosphate transfection. Lentivirus-containing medium was harvested 48 and 72 h after transfection, centrifuged (800× *g* for 20 min) and filtered (0.45 µm pore size). The virus was subsequently concentrated by mixing the filtered supernatant with 50% PEG-8000 (Sigma) in a ratio of 4:1 and placed at 4 °C overnight. The concentrated viral particles were pelleted by two centrifugation steps (2500 rpm for 20 min, followed by 1200 rpm for 5 min), resuspended in 200 µL of PBS and stored at −80 °C.

### 2.3. Generation of TRKA-Tomato Reporter hESC Line

A *TRKA*-tomato reporter hESC line was generated using homologue recombination with the help of CRISPR/Cas9 technology. To this end, a targeting construct was cloned containing a tandem tomato sequence linked via a T2A sequence to the 5′ homology arm containing parts of *TRKA*-Exon 17, as well as to a 3′ UTR site. The vector also included a blasticidin selection cassette. *TRKA*-specific target sites for CRISPR-Cas9 were designed using the CRISPR design online tool from either DNA2.0 (now ATUM) or from the Jack Lin CRISPR/Cas9 gRNA finder. Two oligos were used: oligonucleotides score 1: CACCGCTGGGCTAGGGGGCCGGCCC-forw; AAACGGGCCGGCCCCCTAGCCCAGC-rev and oligonucleotides score 2: CACCGCCTGGATGTCCTGGGCTA-for; AAACTAGCCCAGGACATCCAGGC-rev. These were cloned into CRISPR:hSpCas9 (px330, Adgene) and used for nucleofection. To this end, a single-cell suspension of hESCs was prepared using EDTA and thorough pipetting. Cells were spun at 1000 rpm and counted. 8 × 10^5^ cells were resuspended in 100 μL Ingenio-solution (Mirrus) supplemented with ROCK inhibitor (10 μM, Y-27632, Stem Cell Technologies, Vancouver, Canada) and 2.5 μg of CRISPR:hSpCas9 plasmid and 2.5 μg linearized DNA targeting plasmid and immediately pulse with program DC100 (using a 4D-Nucleofector, Lonza, Basel, Switzerland). Cells were resuspended in warm mTeSR1 (Stem Cell Technologies) supplemented with ROCK inhibitor (10 μM) and plated on one Matrigel coated 10 cm dish. Blasticidin selection (2 μg/mL, InvivoGen, San Diego, CA, USA) was started 2–4 days after plating of cells and picking of clones was started 10–14 days after nucleofection. Picked clones were plated on Matrigel-coated 96-well plates in the presence of ROCK inhibitor. ROCK inhibitor was left out 1–2 days after picking. For Southern blot analysis, DNA was isolated from 96 well plates. Several positive clones for *TRKA*-tomato hESCs were derived and tested for their differentiation potential. The same strategy was used to generate a *TRKA*-tomato *PIEZO2^–/–^* hESC line, using the previously generated *PIEZO2^–/–^* hESC line^2^ as starting material.

### 2.4. Primary Cultures of DRG Neurons

All animal work was approved by the Ethics and Animal Welfare Committee of the University of Heidelberg and the State of Baden-Württemberg or the National Institutes of Health (NIH) guidelines approved by the National Institute of Neurological Disorders and Stroke (NINDS) Animal Care and Use Committee. C57BL/6 female mice at 6 weeks of age were sacrificed using isoflurane and subsequent decapitation. DRGs from all spinal segments were collected in Ringer solution. DRGs were subsequently treated with collagenase IV (1 mg/mL; Sigma-Aldrich) and trypsin (0.05%, Invitrogen, Waltham, MA, USA) at 37 °C for 60 min and 15 min at 37 °C, respectively. After enzymatic treatment, DRGs were washed twice with growth medium [DMEM-F12 (ThermoFisher Scientific) supplemented with L-glutamine (2 µM; Sigma-Aldrich), Antibiotic-Antimycotic (1x; ThermoFisher Scientific) and 5% fetal calf serum (Invitrogen)], triturated using a 1 mL pipette tip and plated in a droplet of growth medium on a glass coverslip precoated with poly-L-lysin (20 µg/cm²; Sigma-Aldrich) and laminin (4 µg/cm²; Invitrogen). To allow neurons to adhere, coverslips were kept for 1 h at 37 °C in a humidified incubator before being flooded with fresh growth medium. Cultures were used for Ca imaging experiments on the 3rd day after preparation.

#### Primary Cultures of DRG Neurons from Wild-Type and Piezo2 Knockout Mice

Trpv1-Cre::piezo2fl/fl were bred by crossing the Piezo2^fl/fl^ [15] with Trpv1-IRES-Cre animals [16]. Dorsal root ganglia (DRGs) cultures were made from adult Piezo2^fl/fl^ : Trpv1-IRES-Cre or Trpv1-IRES-Cre mice. Animals were euthanized and DRGs harvested in accordance with the National Institutes of Health (NIH) guidelines approved by the National Institute of Neurological Disorders and Stroke (NINDS) Animal Care and Use Committee. For enzymatic, dissociation the ganglia were incubated and gently agitated at 37 °C in collagenase (1 mg/mL in PBS, Sigma-Aldrich, St. Louis, MO, USA) for 5–20 min. The collagenase solution was then removed and replaced with Trypsin (0.25%, ThermoFisher, Waltham, MA, USA) for 2–10 min. Following the enzymatic treatment, the Ganglia were triturated with a fire-polished Pasteur pipette in culture media (88% MEM (Gibco, Gaithesburg, MD, USA), 10% Fetal Horse Serum (Gibco), 1% Pen/Strep (Lonza, Basel, Switzerland), 1% Vitamin mix (Gibco)). The supernatant was then transferred to a 15 mL tube (Corning, Tewksbury, MA, USA) containing 6 ml of culture media and centrifuged for 10 min at 150 g to concentrate the dissociated sensory neurons into a pellet. The supernatant was removed, and the pellet was resuspended in a mixture of 100 ul of culture media and 5 ul of a reporter virus (pAAV-CAG-LSL-td-tomato (cat no. 100048, University of Pennsylvania viral core, Philadelphia, PA, USA)). A 10 ul drop of the cell suspension was then plated onto Poly L-Lysine/Laminin coated coverslips (NeuVitro, Vancouver, WA, USA) in 24-well plates (Corning). The plates were placed in an incubator (37 °C/5% CO_2_) and, after an hour of incubation, 500 ul of the culture media was added into each well. Coverslips of sensory neurons were used for whole-cell recording experiments 36–72 h after plating.

### 2.5. Immunocytochemistry

Cells were fixed in 4% PFA for 10 min at room temperature and washed three times with PBS, before blocking solution (10% goat serum (PAN Biotech) in PBS) was added for 1 h at room temperature. Cells were incubated in primary antibodies solution (antibody diluted in PBS containing 3% goat serum, 0.01% Triton x-100 (Merck)) at 4 °C overnight. Cells were washed three times with PBS and secondary antibodies and DAPI (1:12,000) were added to the cells in PBS containing 3% goat serum and 0.01% Triton X-100 for 2 h at room temperature in the dark. Cells were washed three times with PBS and mounted on glass slides with self-made mowiol. Stainings were dried at room temperature overnight in the dark and kept on 4 °C further on.

Primary antibodies used: Chicken anti-Neurofilament 200 (1:25,000; Abcam, #ab72996), mouse anti-ISL1 (1:100; Developmental Studies Hybridoma Bank, #39.4D5), rabbit anti-MAFA (1:20,000; obtained from Dr. Carmen Birchmeier, Berlin, Germany). Alexa 488-, 555- and 647-conjugated secondary antibodies were obtained from Molecular probes and used 1:1000.

### 2.6. In Situ Hybridization Combined with Immunocytochemistry

In situ hybridization was carried out as previously described [17] and optimized for human cells. HESC-derived neurons were fixed for 30 min in 4% PFA, acetylated and permeabilized in 0.3% TritonX–100 in PBS. After pre-hybridization, hydrolyzed DIG- and/or FITC-labelled RNA probes were added, and hybridization was performed overnight at 60 °C. The next day, cells were washed twice with 2 × SSC/50% Formamide/0.1% N-Lauroylsarcosine at 60 °C and then treated with 20 µg/mL RNase A for 15 min at 37 °C. After washing twice in 2 × SSC/0.1% N-Lauroylsarcosine and twice in 0.2 × SSC/0.1% N-Lauroylsarcosine at 37 °C for 20 min each, cells were blocked in MABT/10% goat serum/1% blocking reagent.

For a double fluorescent in situ hybridization, sections were stained by two sequential rounds of Thyramide signal amplification (TSA) steps with an intermediate peroxidase inactivation with 3% H_2_O_2_ for 2 h and 4% PFA for 30 min. Tissue was incubated with anti-FITC-POD (1:2000; Roche) or anti-DIG-POD (1:1000; Roche) overnight at 4 °C. After washing in MABT, the TSA reaction was performed by applying either Thyramide-Biotin on the third or Thyramide-Rhodamine on the fourth day for 30 min at room temperature. For the detection of Thyramide-Biotin a streptavidin-cy2 antibody (1:1000; dianova) was applied, whereas nuclei were stained with DAPI. Subsequently, cells were washed and blocked in 10% NGS in PBS for 1 h, before immunostaining was started as described above.

In situ probes for *PIEZO2* and *RET* were obtained from Dr. Hagen Wende (Institute of Pharmacology, University of Heidelberg; Heidelberg, Germany). Full-length clones for human *TRPV1* and *TRPA1* were provided by Dr. David Julius (University of California, San Francisco, CA, USA), and the generated in situ probes covered the complete open reading frame. The remaining in situ probes were amplified using the following primers and cloned into pBluescript SK(+).

*NTRK1 (TRKA):* (Fwd primer 5′–3′) gacctcgagTCTGGAGCTCCGTGATCTGA, (Rev primer 5′–3′) gacgcggccgcCCGTTGTTGACGTGGGTG; *NTRK2 (TRKB)* (Fwd primer 5′–3′) attactcgagTGGAGCCTAACAGTGTAGATCCTGAGAAC, (Rev primer 5′–3′) atatgcggccgcTGGTACTCCGTGTGATTGGTAACATG; *NTRK3 (TRKC)* (Fwd primer 5′–3′) attactcgagTGGATGTCTCTCTTTGCCCAGC, (Rev primer 5′–3′) atatgcggccgcATTCACCAGCGTCAAGTTGATGG.

### 2.7. Imaging and Quantification

Fluorescence images were taken on a Zeiss Axio Observer A.1 inverted microscope using a Cool Snap camera from Visitron Systems or a Nikon Eclipse Ts2 microscope.

In situ texting and immunocytochemistry for the indicated markers were performed on cells of at least three independent differentiations. For quantification, cells were stained after 21–26 days in culture and 4–5 pictures from randomly selected areas of each staining were taken with the microscope. Cells positive for the respective markers were counted using ImageJ software.

### 2.8. RNA-Seq Sample Generation and Anaylsis

For LTMRs, RNA-sequencing data were taken from Schrenk–Siemens et al., 2015. For NOCL1 and NOCL2, clumps of 10–15 cells were picked individually and amplified using the Smart-Seq2 protocol [18].

For NOCL3, RNA was isolated at different time points (0, 12, 24 and 31 days after induction of *NGN1*) from whole 3.5 cm dishes using the Trizol reagent (Thermo Fisher scientific), based on Chomczynski and Sacchi, 1987 [19]. Integrity of the extracted RNA was investigated, using the Agilent RNA 6000 Bioanalyzer Nano Kit (nCounter Core Facility, Institute of human genetics, University Heidelberg). Libraries were generated using the Illumina TruSeq mRNA library prep kit. Samples were run on an Ilumina NextSeq 500 sequencer on paired-end mode with 75 bp (Genomics Core Facility, EMBL, Heidelberg). Fastq files were aligned using the GRCh38.p12 assembly transcriptome acquired from GENCODE and the Salmon pseudoalignment package [20]. Quality control was performed using FastQC and MultiQC [21,22]. Alignments were imported into R using the tximport package and normalized counts calculated using DESeq2 [23,24]. Data was deposited on ArrayExpress under the following accession numbers: E-MTAB-12098 and E-MTAB-12099.

Single-cell RNAseq data were downloaded from GEOBUS (GSE168243) and the associated cell labels were provided by the authors upon request [25]. Spatial transcriptomic data and the spot identities were downloaded from a publicly available platform provided by the authors (https://sensoryomics.shinyapps.io/RNA-Data/ accessed on 17 September 2022, [26]). Raw counts from both methods which were used as reference datasets for BayesPrism [27] combined with raw counts from either LTMR, Dox (on/off) or NOCL3 Bulk RNAseq datasets.

### 2.9. Ca^2+^-Imaging

HESC-derived nociceptor-like cells, LTMRs or mouse DRG neurons were loaded with FURA-2 AM (10 μg/mL, HelloBio, Bristol, UK) or Cal520 AM (5 μM, AAT Bioquest) into Ringer [(mM): 140 NaCl, 5 KCl, 2 CaCl_2_, 2 MgCl_2_, 10 HEPES and 10 glucose, adjusted to pH 7.4] with pluronic acid, F-127 (0.5 μg/mL; ThermoFisher Scientific) for 1 h at room temperature or 37 °C respectively, in the dark. Cells were washed twice with Ringer and kept at room temperature for at least 20 min before starting the experiments. Fluorescent images were acquired with Metaflour Software (Molecular Devices, San Jose, CA, USA), traces extracted using Suite2p and analysed with image J and/or Graph Pad Prism Software. For standard experiments, cells were challenged with Capsaicin (1 μM, Tocris), Menthol (500 μM; Sigma) or Mustard Oil (Allyl-isothiocyanate, 200 μM; Sigma). For sensitisation experiments, cells were challenged 5 times for 1 min with Capsaicin (10 or 100 nM), with 5 min of washing with Ringer in between. After the 5th stimulation, cells were incubated for 5 min with either NGF (nerve growth factor, 100 ng/μL; Peprotech), PMA (phorbol 12-myristate 13-acetate, 1 μM; Sigma), Bradykinin (10 nM; Tocris) or Serotonin (100 μM, Tocris), followed by another stimulus of Capsaicin (10 or 100 nM) for 1 min. To assess the amount of Capsaicin responders in general, another Capsaicin challenge (1 μM) was given after 5 min washing. For all Ca-imaging experiments, cells were challenged with high K^+^ Ringer solution [(mM): 45 NaCl, 100 KCl, 2 CaCl_2_, 2 MgCl_2_, 10 HEPES and 10 glucose, adjusted to pH 7.4] at the end to visualize all neurons in the culture. For analysis, the average fluorescence ratios (F_340_/F_380_) for capsaicin only, mustard oil only, mustard oil and capsaicin or high potassium only were calculated and shown as traces or parts of whole tables.

For the analysis of the sensitization experiments, the ratio of calcium responses between peaks 6 and 4 (second peak before and first peak after incubation with NGF, PMA, serotonin or bradykinin) was compared.

To test the temperature response of the NOCL3 in comparison to mouse DRGs, temperature challenges of 25 s duration were given to the cells, with 5 min intervals at room temperature in between. Different temperature stimuli were generated using a self-made glass coil system connected to a water bath, temperature at the cells was measured using a temperature probe (physitemp) and increased temperature steps (starting at 35 °C, until 48 °C) were used. Slight variations in the temperature at the side of the measurement could be observed due to thermal bridges in the system. To assess TRPV1-positive cells and neurons in general, a capsaicin stimulus (1 μM) and high potassium stimulation, respectively, were given after the last temperature stimulus. An acquisition rate of 10 Hz was used in combination with the calcium dye Cal520-AM. Cells were segmented and traces extracted using the Suite2p pipeline [28]. R was used to calculate the dF/F0 ratios that were then used to generate the heatmaps using the ComplexHeatmap package [29].

### 2.10. Electrophysiological Recordings

#### 2.10.1. Patch Clamp Recordings

Cell culture medium from cells cultured on 3.5 cm dishes was exchanged with a ~4 mL pre-warmed (37 °C) external solution consisting of (in mM) 136 NaCl, 4 KCl, 2 CaCl_2_, 1 MgCl_2_, 10 D-glucose, 10 HEPES, adjusted with NaOH to pH 7.4, resulting in ~295 mOsm. Using an inverted microscope (Zeiss, Axio Observer A1), differential interference contrast or phase contrast light images were acquired with a 20X objective. Red fluorescence was tested with a HXP 120 C light source and a mCherry filter set. Whole-cell patch clamp recordings were performed with an Axopatch 200B amplifier, sampled at 20–100 kHz, low-pass filtered at 10 kHz and digitized with a Digidata 1440A using Clampex 10.6. Recordings were performed at room temperature (22–24 °C) up to 4–6 h with borosilicate glass (O.D. 1.5 mm, I.D. 0.86 mm, Sutter Instrument, BF150-86-7.5), pulled on a micropipette puller (P-97, Sutter Instrument) with an open pipette resistance of ~2–4 MΩ measured with a KCl- or CsCl-based patch pipette solution, subsequent gigaseal >1 GΩ. Patch pipettes were filled with (in mM) 1 EGTA, 10 HEPES, >115 KCl, 6 MgCl_2_, 6 Na_2_-phosphocreatine, 4 Na_2_-ATP, 0.3 Na-GTP, pH-adjusted to 7.4 with KOH and osmolarity-adjusted with further addition of KCl to 280–285 mOsm. Neurons were held at −65 mV in a voltage clamp.

Cell capacitance was assessed in a voltage clamp by hyperpolarizing the cell by 15 mV for 100 ms, averaging 25 response current traces and fitting a biexponential decay to baseline to the first capacitance decay. Cell capacitance was calculated by dividing the weighted biexponential decay by the cell’s input resistance. The cell’s input resistance was calculated by dividing the applied voltage step by the relative current of the first capacitive transient.

Action potentials were measured in current clamp with active bridge balance. Cells were held at −65 mV by injecting current. Increasing current steps of 100 ms or 1 s were applied to evoke action potentials.

The occurrence of a ‘shoulder’ in the falling phase of the action potential was quantified by fitting a monoexponential function between the peak of the action potential and the minimum of its afterhypolarization (AHP), assessed as the voltage minimum within 20 ms after the peak of the action potential.

The rising of the after-hypolarization (τ_AHP_) was assessed by a monoexponential fit of 32 ms length, starting at the minimal voltage within 20 ms after the action potential peak, if the AHP amplitude was at least 0.1 mV and no second action potential was elicited.

Mechanically activated currents were recorded at −65 mV with a heat-polished glass pipette of the same properties as used for the patch clamp, attached to a micromanipulator (Nanomotor, MM3A, Kleindieck Nanotechnik, Reutlingen, Germany). The evoked whole-cell currents were sampled at 20–100 kHz and hardware (Axopatch 200B) low-pass filtered at 10 kHz and subsequently software low-pass filtered with 1 kHz. The micromanipulator probe was positioned at an angle of 45° to the surface of the dish at opposing site to the patch pipette. The probe moved with a velocity of ∼1.3 µm/ms. Currents were fit by a bi-exponential function to the baseline before the stimulus and classified as rapidly adapting (<10 ms), intermediate-adapting (10–30 ms) and slowly adapting (>30 ms) mechanical currents according to their weighted biexponential inactivation time constant. Cells were classified as mechanically inactive when stimuli of ≤10 µm indentation did not elicit responses >−40 pA. Recordings in which leak currents significantly increased upon mechanical stimulation were not considered.

Amplitudes of mechanically evoked currents were either determined by the weighted amplitude of the biexponential fit or by measuring the relative amplitude.

For assessment of TTX-resistant sodium currents, a diamond-shaped recording chamber was constantly perfused (∼2 mL/min) with a gravity-driven ValveLink 8.2-system (AutoMate Scientific, Berkeley, CA, USA) with (in mM) 128 NaCl, 20 TEA-Cl, 1 4-AP, 1 CaCl_2_, 1 MgSO_4_, 8 glucose, 10 HEPES, 0.1 CdCl_2_, adjusted to pH 7.4 with NaOH, resulting in ~300 mOsm adding Tetrodotoxin citrate (HB1035, Hello Bio, Bristol, UK or cat. no. 1069, Tocris). Patch pipettes were filled with (in mM) 10 EGTA, 10 HEPES, >100 CsCl, 6 MgCl_2_, 4.8 CaCl_2_, 6 Na_2_-phosphocreatine, 4 Na_2_-ATP, and 0.4 Na-GTP; pH was adjusted with CsOH to 7.4; osmolarity was adjusted by further addition of CsCl to ~280–285 mOsm. In the voltage clamp, with actively compensated pipette series resistance and whole-cell capacitance (~70%), cells were held at −65 mV. They were then hyperpolarized for 200 ms to −120 mV to recover TTX resistant currents, and then clamped for 40 ms to potentials from −50 mV to 50 mV in steps of 5 mV.

Analysis was carried out in Igor Pro 6.37 (WaveMetrics, Tigard, OR, USA), after importing Axon binary files with DataAccess (Bruxton Corporation, Seattle, WA, USA), or in Graphpad Prism 5, 7 or 8. A two-sided Mann–Whitney statistical test was performed, as indicated in the respective legends. Adjustment for multiple comparisons was not performed.

#### 2.10.2. Neuronal Voltage Clamp Recording of Mouse DRG Cells

Whole-cell current responses of intersectionally targeted Trpv1 and td-tomato-expressing neurons were recorded. Recordings were done using a Multiclamp 700B amplifier (Molecular Devices, San Jose, CA, USA) at a holding potential of −60 mV. Signals were digitized with a Digidata 1550 (Molecular Devices) digitizer at 100 kHz, low-pass filtered at 10 kHz, and saved on a PC computer running Clampex 10.4 (Molecular Devices). The extracellular solution used consisted of 133 mM NaCl, 3 mM KCl, 1 mM MgCl2, 10 mM HEPES, 2.5 mM CaCl2, 10 mM glucose, and 18.9 mM sucrose (pH 7.3 with NaOH, OSM = 302). The patch pipettes (resistance of 3–10 MΩ) were filled with an intracellular recording solution consisting of 133 mM CsCl, 10 mM HEPES, 5 mM EGTA, 1 mM CaCl2, 1 mM MgCl2, 4 mM Mg-ATP, 0.4 mM Lithium-GTP, and 10 mM Cs-gluconate with CsOH (pH 7.3 OSM = 285).

Mechanical activation of dissociated sensory neurons was performed by driving a heat-polished blunt pipette tip (3–5 mm) at an approximately 60° angle into the center of the cell using a micromanipulator mounted on a P841.20 piezoelectric translator (Physik Instrumente, Karlsruhe, Germany). A series of 10 increasing indentations (1 μm increments) was applied to each cell. Cells that detached from the surface of the coverslip or lost the patch before the entire series of indentations was applied were excluded. Cells were identified as responding only if the maximum evoked current exceeded 50 pA. Fisher’s exact test with confidence level of 0.05 was used to test significance.

## 3. Results

### 3.1. Generation of hESC-Derived TRKA-Positive Neurons

The diverse spectrum of sensory neurons emerges from a transient cell population during development: the neural crest cells, which serve as progenitors for the sensory neurons located in the dorsal root ganglia (DRG). Early instructive roles for sensory neuron differentiation by Neurogenin1 (NGN1) and Neurogenin2 (NGN2), two transcription factors expressed in sensory lineage-primed neural crest cells, are well-documented [7]. While the majority of large diameter TRKB- and TRKC-positive sensory neurons primarily depend on NGN2, the generation of small diameter neurons requires NGN1 expression. It is this latter group that comprises nociceptors, sensory neurons specialized for detecting painful—often considered harmful—stimuli.

**Figure 1:**
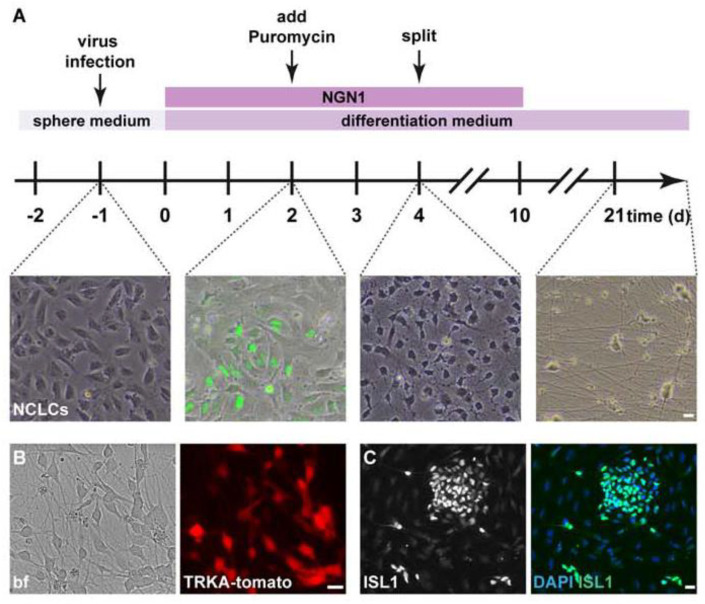
(**A**) Schematic illustration of the differentiation protocol to generate hESC–derived nociceptor–like cells: arrows indicate time points of virus infection, selection (addition of puromycin) and splitting of cells. Graphs show characteristic morphology of cells at the indicated time points: –1 day (d) neural crest–like cells (NCLCs), 2 d NGN1 expression, indicated by GFP–signal; 4d neural progenitors show small neurites; ≥21d neurons with long processes can be observed. (**B**) Live cell imaging of neuronal progenitors derived from the hESC TRKA–tomato reporter cell line: left picture shows a bright field (**bf**) image, right picture the tomato signal of cells 6 days after doxycycline–induced expression of NGN1. (**C**) Immunostaining of hESC–derived NCLCs 2 days after induction of NGN1–expression. As early as 2 days after NGN1 induction, the presence of the pan–sensory marker ISLET1 (ISL1) is detectable in a subset of cells. (DAPI: blue; ISL1: green). Scale bars: 20 μm.

We made use of this defining feature and transiently expressed NGN1 in hESC-derived neural crest-like cells (NCLCs), a cell population that we and others have previously shown to inherently hold the potential to become sensory-like neurons [12,14]. In order to drive and control NGN1 expression, NCLCs were infected with two lentiviruses (Figure 1A and Figure S1A): one lentivirus expressed a tetracycline-controlled transactivator (rtTA) under the control of a ubiquitin promoter for constitutive expression of rtTA. The second lentivirus expressed NGN1, EGFP, and a puromycin resistance cassette linked with P2A and T2A sequences, respectively. This took place under the control of the tetracycline operator (tetO) promoter. By adding doxycycline (Dox) to the culture medium, the expression of NGN1 was induced and verified by the presence of EGFP. Thereby, we were able to reversibly control the duration of NGN1 expression (Figure 1A and Figure S1B). Interestingly, after switching viral NGN1 expression off, NGN1 transcription initially declined, but stayed elevated in comparison to baseline levels. Notably, NGN2 was not detectable at any time point (Figure S1C). Presumably, the endogenous NGN1 gene (but not the NGN2 gene) was activated in differentiating cells.

One to two days after onset of exogenous NGN1 induction, the NCLCs began to change their morphology and islands of roundish EGFP-positive cells, encircled by elongated, and EGFP-negative cells could be observed (Figure 1A). To eliminate non-infected (EGFP-negative) cells and to enrich NGN1-expressing NCLCs, puromycin was added for up to 48 h into the Dox-containing culture medium. Four to five days after induction of NGN1 expression, neuronal progenitors with small neurites were observed (Figure 1A), most, if not all, expressed NGN1/EGFP (data not shown).

In vivo, NGN1 is transiently expressed in neurons that will express the receptor tyrosine kinase TRKA, a gene that marks nociceptive sensory neurons [30]. We therefore limited expression of NGN1 to the first 10 days of differentiation (Figure 1A). Morphological features alone are insufficient to unambiguously demarcate nociceptive neurons into mixed neuronal cultures. Therefore, we generated an hESC reporter line, which expresses td-tomato under the control of gene-regulatory elements of the TRKA receptor. The TRKA gene was left intact and the tomato sequence was introduced before the stop codon of the TRKA locus with a T2A linker sequence using CRISPR/Cas9 technology (Figure S2A,B). The fluorescent reporter allowed us to follow the in vitro differentiation of nociceptive neurons by microscopy: six days after NGN1 induction, the first tomato-positive cells could be observed when using the TRKA reporter cell line (Figure 1B). The tomato-positive neurons started to develop processes shortly after NGN1 induction and continued to generate an elaborate network of neuronal processes in the course of the culturing period (Figure 1A). Two days after NGN1 induction, the NCLCs started to express ISLET1 (Figure 1C), an indication that the cells had begun to acquire sensory neuron-like states. Puromycin selection yielded sensory neuron cultures of high purity (84.6 ± 12.6% after 21–25 days in vitro (div); 90.9 ± 4.6% after 42–58 div). Cultures not virally induced to express NGN1 did not express the fluorescent TRKA reporter (data not shown). To further assess the validity of the TRKA reporter and to verify co-expression of the fluorescent tomato transgene, together with endogenous TRKA transcripts, double fluorescence in situ hybridizations were performed. These experiments demonstrated high (close to 100%) overlap of tomato and TRKA expression (Figure S2C).

Collectively, these results show that driving expression of NGN1 in hESC-derived sensory neuron precursors (NCLCs) is sufficient and highly efficient in generating TRKA-positive **no**ci**c**eptor-**l**ike neurons that from here on we dub NOCL neurons.

### 3.2. Functional Characterization of TRKA-Positive Neurons

Mature nociceptors are specialized to detect a variety of disparate physical (mechanical and thermal) as well as inflammatory stimuli, which accounts for differences in their molecular armament [31,32]. These differences likely are the result of developmentally induced differences in their transcriptional programs [33]. Previous attempts to generate nociceptors have shown that it is difficult to efficiently produce discrete nociceptor sub types [3,4,5]. We therefore wondered whether recapitulating key steps of nociceptor development—instead of trans-differentiating hESCs or fibroblasts directly into nociceptors as employed in previous protocols—would create a permissive environment to yield homogenous populations of discrete nociceptor subtypes. With this goal in mind, we first generated neural crest-like cells (NCLCs), altered their internal transcriptional program and varied the composition of external (neurotrophic) factors.

**Figure 2:**
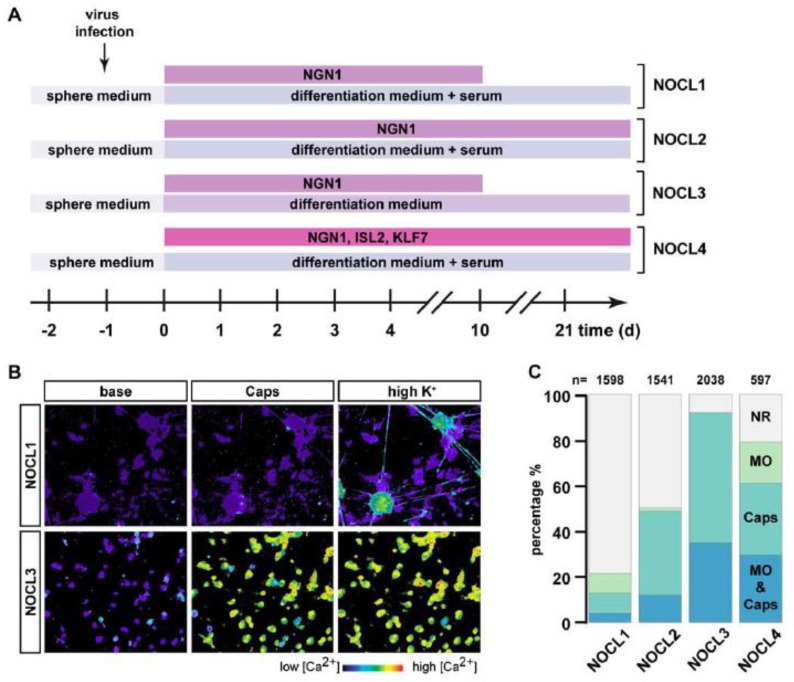
(**A**) Schematic illustration of the differentiation procedures used to generate different types of hESC–derived nociceptor–like cells (NOCL1–4), showing the differences in medium composition (±serum), the type of transcription factors used (NGN1, ISL2 and KLF7) and the duration of their expression (10 days or until the end of the culturing period). (**B**) Representative pseudo–colored images of calcium responses observed in NOCL1 and NOCL3 cells loaded with Fura2. Shown are the respective Ca2+ levels before (base) and during stimulation, with either the TRPV1 agonist capsaicin (Caps, 1 μM) or high K^+^ Ringer solution for general depolarization of neurons. (**C**) Stacked bar charts, show the percentage of NOCL1–4 cells responding to mustard oil (MO, 200 μM), capsaicin (1 μM), or both agonists using calcium imaging. (*n* = 4). Data shown as average percent ± SEM.

During mouse DRG development, NGN1 is transiently expressed. However, it is unclear at what time point during human DRG development NGN1 is turned on and for how long its expression persists. We therefore compared conditions of transient and extended NGN1 expression, designated NOCL1 and NOCL2, respectively (Figure 2A). Additionally, external factors such as neurotrophins have been shown to influence sensory neuron fate. We therefore included a culturing condition devoid of serum; instead, we used a defined medium only containing neurotrophins implicated in sensory neuron differentiation (NOCL3—Figure 1A and Figure 2A; see methods for details). Last but not least, we also modified a previously published differentiation protocol and included two additional transcription factors KLF7 and ISL2—instead of NGN1 alone—which had been shown to induce nociceptor-like cells [5]. Different to the previous protocol, which uses human fibroblasts or embryonic stem cells as starting cell population, we introduced these factors into derived NCLCs, based on the assumption that the native precursors of nociceptors are more readily primed to differentiate into sensory neurons. We refer to this differentiation procedure as NOCL4 (Figure 2A). Next to te TRKA expression, a hallmark of many nociceptors is the expression of the capsaicin receptor TRPV1 [1]. Thus, a common way to identify and characterize nociceptors is to test their response to capsaicin, the pungent ingredient of chili peppers that triggers the opening of the TRPV1 ion channel to permit cation permeation such as the influx of calcium ions. Accordingly, following a differentiation period of at least 3 weeks, we evaluated the differently generated NOCLs for their ability to respond to capsaicin. NOCL cultures were loaded with the calcium indicator Fura-2 and challenged by 1 μM capsaicin. Remarkably, we found that the NOCL cultures differed considerably in their response to Capsaicin: while only 13 ± 2% of neurons responded to capsaicin when NGN1 was turned off after 10 days (NOCL1 differentiation protocol; Figure 2B,C), expressing NGN1 constitutively for the entire differentiation period resulted in 48.9 ± 7.6% of capsaicin responders (Figure 2C).

Cultures derived using a cocktail of virally expressed transcription factors NGN1, ISL2, and KLF7 [5] (NOCL4) produced 61.1 +/− 14.7% capsaicin responsive neurons (Figure 2C). Strikingly, limiting NGN1 expression to 10 days and nurturing differentiating neurons in a defined culture medium absent of any serum (NOCL3—see methods for details) resulted in a nociceptor-like cell population that homogenously responded to capsaicin: We found that most if not all derived neurons responded to capsaicin (92.2 ± 5.9%). This is in stark contrast to low-threshold mechanoreceptors (LTMRs), generated using our previously published protocol [12], which did not yield any TRPV1-expressing neurons, and that here served as a control cell population (Figure 2B,C and Figure S3A).

The ability to efficiently generate TRPV1/TRKA-positive NOCL neurons was specific for NGN1 and could not be recapitulated by viral expression of either RUNX1 (Figure S3B) or TRKA (Figure S3C), two genes that are induced subsequent to NGN1 expression during murine nociceptor development [33].

In native mouse and human DRGs, another TRP ion channel, TRPA; this is expressed in a subset of TRPV1-positive neurons [34]. Interestingly, the differentiation protocols also differed considerably in the fractions of TRPA1-postive neurons, as assessed by calcium imaging using the TRPA1 agonist mustard oil: NOCL1 neurons showed only a small population of TRPV1 cells responding to mustard oil (4.2 ± 1.6%) (Figure 2C). In NOCL2, NOCL3 and NOCL4 cultures, fractions of 12.1 ± 4.3 %, 35.1 ± 16.6% and 29.7 ± 8.2% of capsaicin-responsive cells responded to mustard oil, respectively (Figure 2C). Almost none of the cultures harbored any menthol-responsive cells (data not shown). Collectively, these results show that the four NOCL differentiation procedures yielded different fractions of sensory TRP channel positive neuronal populations.

### 3.3. Molecular Characterization of TRKA-Positive NOCL Neurons

**Figure 3:**
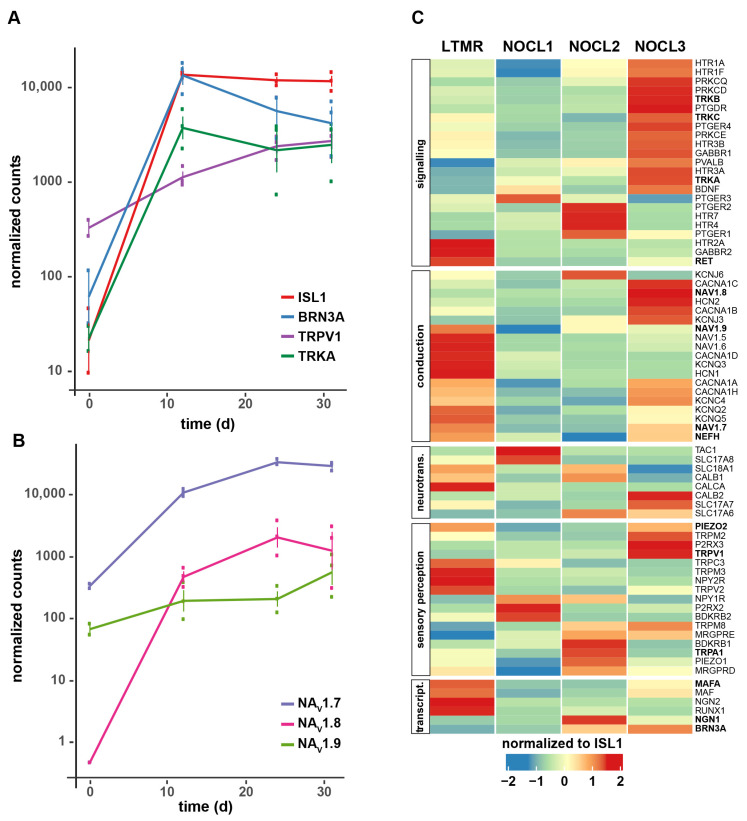
(**A**) RNA–seq analysis of ISL1, BRN3A, TRPV1 and TRKA transcripts in NOCL3 cells at specific time points during their differentiation (0, 12, 24 and 31 d), shown as normalized counts (for analysing details see material and methods; *n* = 3). (**B**) RNA–seq analysis of NA_V_1.7, NA_V_1.8 and NA_V_1.9 transcripts in NOCL3 cells at specific time points (0, 12, 24 and 31 d) during their differentiation, shown as normalized counts (*n* = 3). (**C**) Heatmap showing the relative expression of selected genes in LTMR and NOCL1–3 cells after 24 days of differentiation. Genes are categorized into functionally connected groups, and those indicated in bold have been analysed in more detail (neurotrans. = neurotransmission, transcript. = transcription). Gene counts were first normalized and then related to the pan–sensory marker ISL1 and averaged (*n* = 3). Depicted are center–scaled values.

To gain a more complete picture of the molecular identity of the derived nociceptor cells, we surveyed gene expression at several time points throughout the differentiation period (day 0 –before NGN1-induction–, day 12, day 24 and day 31), focusing on the NOCL3 protocol yielding the highest percentage of TRKA/TRPV1-positive neurons (Figure 2C). We found that NGN1 expression triggered a gene program characteristic of developing sensory neurons, with ISLET1 and BRN3A induced early on during the differentiation process (Figure 1C, Figure 3A and Figure S4). Expression profiling showed that, next to TRPV1, other prototypical nociceptor genes such as NA_V_1.7, NA_V_1.8 and NA_V_1.9 [35] were expressed upon NOCL3 differentiation (Figure 3A,B and Figure S4). We also found strong induction of TRKA expression (Figure 3A and Figure S4), further confirming the reliability of our TRKA-tomato reporter cell line. Transcript levels for most of these nociceptor markers reached a stable expression level between 24 to 30 days of differentiation (Figure 3A,B and Figure S4), suggesting that a steady state was reached.

We wondered whether subtly altering the in vitro differentiation procedure (duration of NGN1 induction, availability of external morphogenetic factors) would change the fate of the ensuing NOCL neurons. Therefore, we compared expression profiles of the different NOCL cultures at 24 days in culture, a time point, where a steady state for several key nociceptive genes was observed (Figure 3A,B).

Indeed, we found that prolonging the expression of NGN1 for the entire duration of the differentiation period (NOCL2) or enriching the medium with serum factors (NOCL1 and NOCL2) had a strong impact on the emerging sensory neurons: short (but not long) NGN1 expression induced the appearance of TAC1 (Substance P), a marker of peptidergic nociceptors, in NOCL1 neurons (Figure 3C). TRPV1, the capsaicin receptor, present in both peptidergic and non-peptidergic nociceptors [31,32,36], was robustly expressed in TRKA-positive NOCL3s. Conversely, it was largely absent from LTMRs and was expressed at a significantly lower level in serum-derived NOCL1s and NOCL2s (Figure 3C), matching the unequal capsaicin responses observed in the different NOCL1-3 cell types (Figure 2B,C).

Other genes of functional relevance for nociceptors and implicated in their sensitization, including G-protein-coupled receptors (e.g., Serotonin-, Prostaglandin- and Histamin receptors), essential signaling molecules (such as PKCε/PKCδ), and P2X2/3- and HCN ion channels were also found to be differentially expressed in NOCL1-3 cultures (Figure 3C).

We found the 3 TRK receptors TRKA, TRKB and—to a lesser extent—TRKC, co-expressed in the NOCL3 neuron population while NOCL1- and 2 neurons were largely devoid of TRKB/C (Figure 3C, Figures S2C and S5A–C). In native murine DRG neurons, conjoint TRK expression is observed during DRG neuron development [30], while in mature sensory neurons, TRKB and TRKC have classically been associated with LTMRs, proprioceptors and other non-nociceptive sensory subtypes. Somewhat surprisingly, we recently reported TRKB and TRKC to be expressed in a small fraction of native TRKA-positive small-diameter neurons in human DRGs (5 and 16%, respectively) [34]. It is therefore possible that the NOCL3 population we describe here either represents a discrete nociceptor subtype that expresses multiple TRK receptors or, alternatively, these cells have not yet reached full maturity.

**Figure 4:**
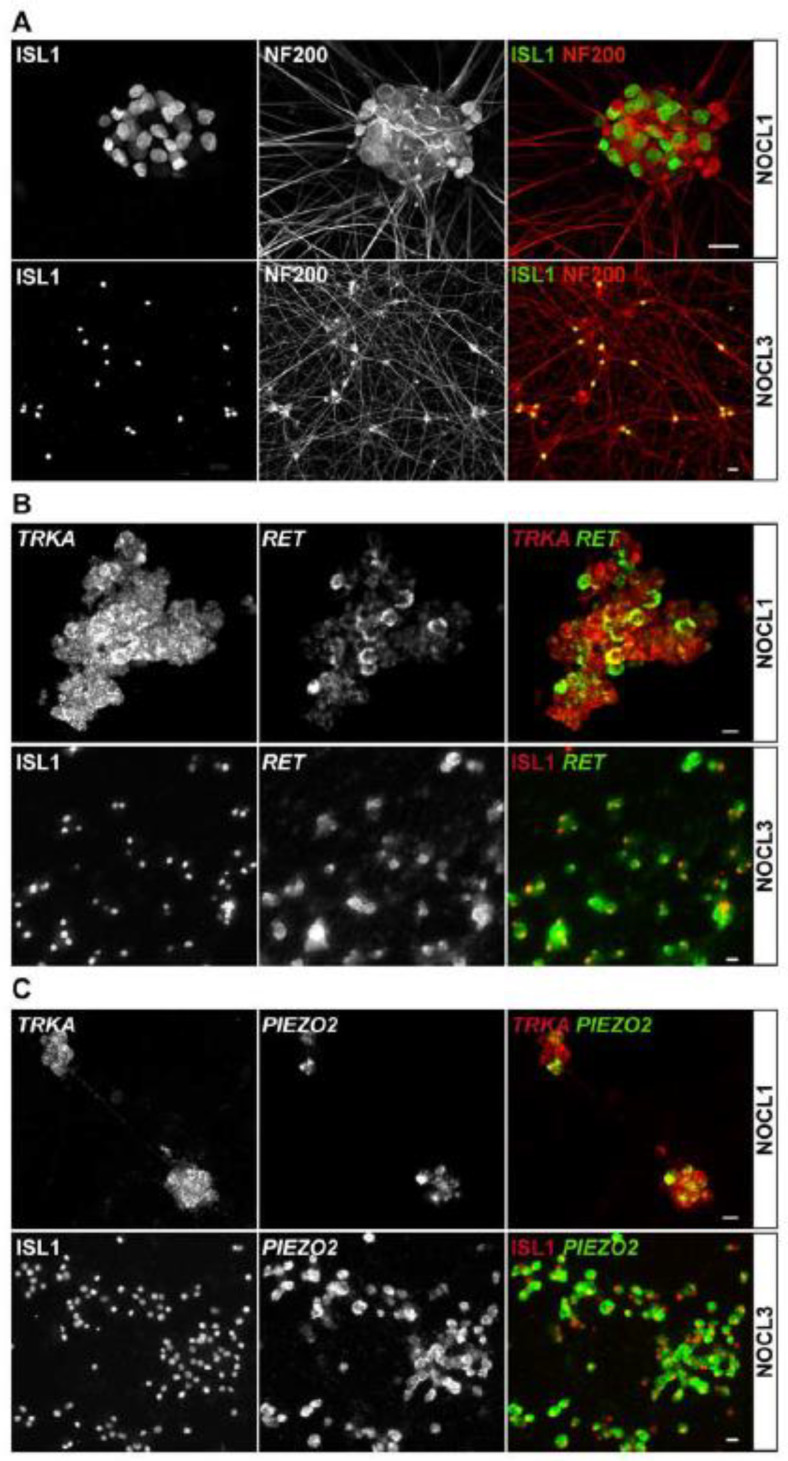
(**A**) Immunostaining of NOCL1 and NOCL3 cells detecting ISL1 protein (**left**), NF200 (**middle**), and the composite image (**right**). Note, the presence of neurofilament heavy 200 is essentially detected in all NOCL cells. (**B**) In situ hybridization of NOCL1 and NOCL3 cells with probes detecting TRKA (upper left and red) and RET (middle and green) as well as immunostaining for ISL1 (lower left and red) to show co-expression of TRKA and RET or ISL1 and RET. In NOCL1 cells 77.6 ± 0.5% of TRKA positive neurons co-expressed RET, compared to 97.4 ± 0.9% in NOCL3 cells (*n* = 3). (**C**) In situ hybridization of NOCL1 and three cells with probes detecting TRKA (upper left and red) and PIEZO2 (middle and green) as well as immunostaining for ISL1 (lower left and red) to show co–expression of the respective markers: 64.2 ± 2.3% of TRKA positive NOCL1 neurons co-expressed PIEZO2, compared to 95.0 ± 2.4% in NOCL3 cells (*n* = 3; mean ± SEM; scale bar 20 μm).

While the level of several transcripts expressed in mature nociceptors plateaued towards the end of the differentiation period (Figure 3A,B), NA_V_1.9, an ion channel associated with pathological forms of pain, had not reached a steady state at the end of the differentiation period but started to increase expression in the 2nd half of the differentiation phase (Figure 3B and Figure S4), providing evidence for the latter possibility and suggesting that NOCL3 neurons are not yet fully mature. It is unclear to what degree insights gleaned from murine models can be extrapolated to human nociceptors. While the overall molecular profile of human and rodent nociceptors is similar, significant molecular differences of discrete nociceptor subtypes have been observed [34]. One prominent difference between native human vs. murine nociceptors is the presence of neurofilament heavy (NEFH, NF200) in most—if not all—human DRG neurons [34], a protein selectively expressed in myelinated murine Aδ- and β-fibers [1]. In agreement with these findings, we find NF200 to be expressed in all NOCL neurons, regardless of the differentiation protocol used (Figure 4A).

The receptor tyrosine kinase RET is robustly expressed in both murine and human DRGs and associated with different classes of sensory neurons. In rodents, RET is expressed in a graded fashion in different subsets of nociceptors, itch-detecting neurons and LTMRs [37] but largely excluded from TRKA-positive neurons [38]. However, in human neurons, RET is differently distributed, with nearly double the fraction of mature human nociceptors co-expressing TRKA and RET (mouse: 23.2 +/− 1.8% vs. human: 45.9 +/− 0.7%, [34,39]. We found RET to be robustly expressed in TRKA-positive NOCL3 neurons (97.4 ± 0.91%), while the receptor was found in 77.6 ± 0.5% in TRKA-positive NOCL1 neurons (Figure 4B). The observed co-expression resembles more closely what has been shown in native human DRG neurons but found only rarely in mouse DRG neurons [34,37].

While RET is widely expressed across small and large diameter neurons, the transcription factor MAFA is a more selective molecular marker for mouse and human LTMRs and largely excluded from nociceptors [17]. We find MAFA transcripts absent from NOCL1 and NOCL2 neurons but detectable in NOCL3 neurons, albeit to a lesser extent compared to LTMRs (Figure S5D). In agreement with a minor—if any—function for MAFA in nociceptors, we find that MAFA protein is only detectable in a few NOCL3 neurons, differing from derived LTMRs which robustly express MAFA (Figure S5E).

Another sensory receptor, PIEZO2, previously identified as the main transducer of mechanical stimuli in LTMRs [11,12], was expressed in NOCL neurons and agreement with data obtained from native mouse and human DRG tissue [34]. All NOCL differentiation procedures yielded PIEZO2-postive neurons, albeit the expression level and the fraction of neurons positive for the ion channel varied (Figure 3C and Figure 4C).

When using recently published sequencing data from human DRGs obtained either by single nucleus sequencing [25] or spatial sequencing [26] to assess cell type composition of NOCL cells, we find our cells to show marker combinations reported for different sensory neuron subtypes present in humans. NOCL3 cells show similarities with Aδ− HTMRs and -LTMRs and pruritogen receptor-enriched neurons, when using the data from Tavares–Ferreira et al., 2022 [26], and with H10 cells (c-type nociceptors) and H12 cells (no mouse counterpart assigned) when using the 2021 dataset of Nyugen et al. [25] (Figure S6A,B). NOCL1 and NOCL2 cells differed considerably when using the Tavares-Ferreira et al. 2022 dataset: while NOCL1 cells have a greater similarity to PENK**^+^** nociceptors and TRPA1**^+^** nociceptors, NOCL2 cells bear similarity to cold nociceptors, a subclass that is neither present in NOCL1 nor NOCL3 cells. This indicates again the differences between the generated sensory neurons in vitro, depending on the duration of NGN1 expression and the exposure to extracellular factors. The influence of NGN1 expression on the assignment of the NOCL3 cells to the respective sensory neuron subgroups described by Tavares–Ferreira et al., 2022, becomes obvious when looking at the course of development of the NOCL3 cells: induction of NGN1 either prevents the generation of certain subgroups such as TRPA1+, putative C-LTMRs or cold nociceptors, while it pushes the generation of pruritogen receptor-enriched cells (Figure S6C). In summary, driving NGN1 expression in human neural crest-like cells combined with exposing them to different extracellular factors promoted the generation of different nociceptor-like cells, with similar molecular profiles compared to subclasses of native human DRG neurons. The lack of assigning the differentiated neurons only to one subclass of sensory neurons could either mean that we do not produce a homogenous population or — more likely—that our cells are not yet fully mature and lack additional factors that help to drive them in a fully developed state.

### 3.4. NOCL3 Neurons Are Heat-Responsive and Can Be Sensitized by Inflammatory Stimuli

**Figure 5:**
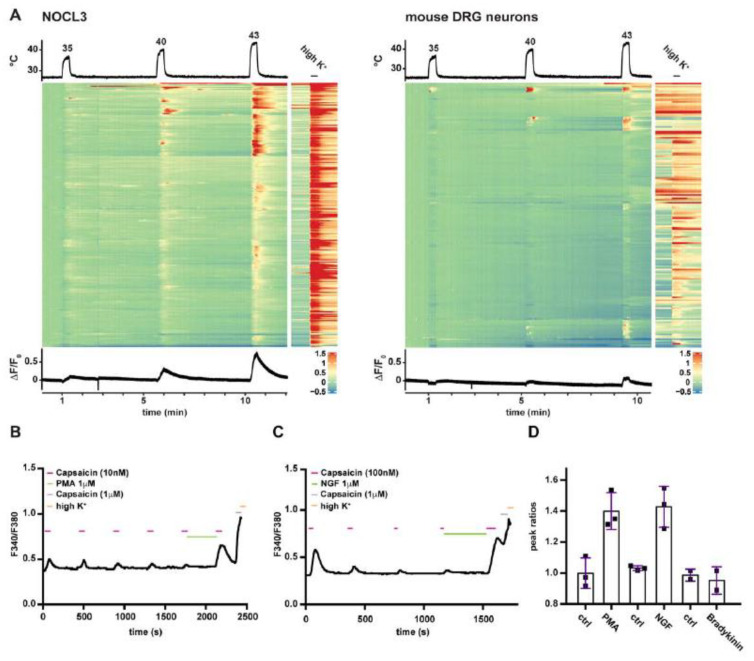
(**A**) Heatmaps of calcium imaging experiments challenging Cal520–loaded NOCL3 and mouse dorsal root ganglion (DRG) neurons with increasing temperatures from 35 °C to 43 °C and subsequently with high K^+^ Ringer solution (to identify all neurons). Every row represents a single cell. Depicted is the ΔF/F_0_ ratio, using the baseline before the first stimulus as F_0_. The trace below the heat map shows the average response of all neurons (NOCL3 *n* = 248 cells; DRG n = 174). (**B**) Representative fluorescence ratios (340 nm/380 nm) obtained from one NOCL3 neuron loaded with Fura2 and stimulated with multiple pulses of capsaicin (10 nM). Incubation of cell with the phorbol ester PMA (1 uM) for 5 min results in sensitization leading to an increased response amplitude in response to a low concentration of capsaicin. Subsequent application of high K^+^ ringer solution showed presence of all functional neurons. (**C**) Calcium response of a NOCL3 neuron stimulated repetitively with capsaicin (100 nM). Incubation with NGF (100 ng/mL) for 5 min sensitized the cell, resulting in an increased response to capsaicin. High K^+^ ringer solution was added at the end to visualize functionality of the neuron. (**D**) Bar graphs represent the ratios between the peak response after and the peak response before application of inflammatory agents as indicated. Fraction of cells showing sensitization: PMA: 91% (753 of 823 cells); NGF: 28% (173 of 611 cells); bradykinin: 0% (0 of 319 cells).

A number of TRP ion channels have been implicated in mediating responses from warm to hot temperatures [40]. In DRG sensory neurons, the most prominent warmth/heat sensitives TRPs are TRPV1, TRPM2, TRPM3, and TRPA1 [41,42,43]. Therefore, we next tested the response of the generated sensory neurons to temperature steps from 35 °C to 43 °C using Ca^2+^-imaging focusing on the population of NOCL3 cells that featured the highest percentage of TRPV1 responders. Different to cultured mouse DRG neurons, hESC-derived neurons responded more homogenously to the temperature stimuli (Figure 5A). These results corroborated uniformity of cells produced by the NOCL3 protocol, resulting in a population of prototypical TRPV1-positive, temperature responsive nociceptors.

Besides responding to heat, the capsaicin receptor can also integrate inflammatory stimuli. Inflammatory sensitization is a characteristic feature of TRPV1. We therefore investigated NOCL3 neurons in the context of inflammatory pain signaling.

Components of the so called ‘inflammatory soup’, a mixture of signalling molecules such as Bradykinin, NGF, Serotonin and others are released at the site of inflammation and activate disparate signalling cascades present in different subsets of nociceptors. These inflammatory cascades converge on TRPV1 and boost channel activity to subthreshold stimuli (primary hyperalgesia) [8].

Sensitization of TRPV1 can be mimicked in vitro by incubating cultured sensory neurons with any of the aforementioned inflammatory mediators. In this experiment, the sensitizing agent is applied in between puffs of low doses of capsaicin [44]. An increase in the magnitude of the calcium influx to the subsequent capsaicin stimulus –compared to the capsaicin response before incubation with the inflammatory mediator—is indicative of TRPV1 sensitization. We tested the ability of the TRPV1-expressing NOCL3 cells to become sensitized by the inflammatory mediators NGF and Bradykinin as well as the phorbol ester PMA. We compared the ratios between the peak response after—and the peak response before application of the respective inflammatory agents and found that hESC-derived nociceptors can be sensitized by the PKC activator PMA (ctrl 1.0 ± 0.1; PMA 1.4 ± 0.12) and the cognate TRKA ligand NGF (ctrl 1.03 ± 0.02; NGF 1.43 ± 0.13), while Bradykinin was not able to sensitize the cells (ctrl 0.99 ± 0.04; Bradykinin 0.95 ± 0.09; Figure 5B–D). This sensitization profile was in agreement with the expression profile of the respective inflammatory receptors determined by transcriptional profiling: while TRKA was present in NOCL3 neurons, the Bradykinin receptor was not detectable (Figure 3C).

Collectively, we conclude that transient expression of NGN1 in hESC-derived NCLCs, cultured in a defined serum-free medium, results in the generation of a molecularly homogenous population of TRKA- and TRPV1-positive population that displays typical features of inflammation-responsive nociceptors.

### 3.5. Electrophysiological Properties of Human Nociceptor-like Cells

**Figure 6:**
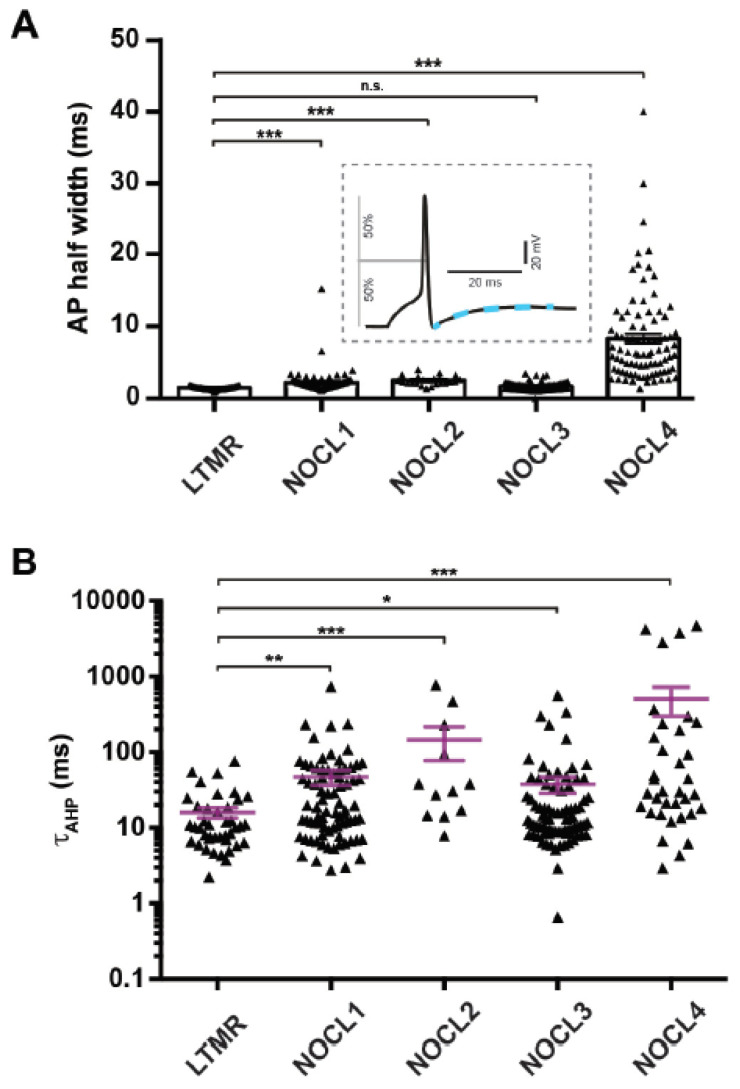
(**A**) Somatic action potential (AP) half-width determined as indicated in the example shown in the inset; LTMR (*n* = 43), NOCL1 (*n* = 109), NOCL2 (*n* = 70), NOCL3 (*n* = 90) and NOCL4 (*n* = 87) were compared by Mann–Whitney test, n.s. (*p* = 0.1243), ** (*p* = 0.0023), *** (*p* < 0.0001). (**B**) Recovery from after-hyperpolarization was fitted with a single exponential function (blue dashed line in (**A**)). n.s. (*p* = 0.1522), * (*p* = 0.0337), ** (*p* = 0.0020), *** (*p* ≤ 0.0003), Mann–Whitney test. In (**A**) and (**B**) each triangle in the scatter plots corresponds to one cell, mean ± SEM is depicted.

We confirmed that all derived nociceptor types, NOCL1 to NOCL4, are excitable and exhibit action potentials (APs) upon current injection, while spontaneous activity could be rarely seen, similar to what is known from primary DRG neurons in culture. Overall, AP half-widths of NOCL1-3 preparations were similar and well in the range of native intermediate-size murine nociceptors that typically give rise to Aδ-fibers [45,46,47] (Figure 6A). AP waveforms of cells derived according to the NOCL4 protocol showed a broader AP width, including a ‘shoulder’ in the AP decay phase (Figure 6A and Figure S7A)—a typical ion current feature found in some human and mouse nociceptors [48]. Additionally, we recorded resting membrane potentials in the range characteristic for small-diameter (C-fiber-type) human and mouse DRG neurons [45] (Figure S7B).

These electrical attributes correlated well with estimated cell sizes assessed by capacitance measurements: the size range of NOCL1-3 neurons was comparable to that of small/intermediate-sized native human nociceptive neurons [34,48]. On average, NOCL4 neurons turned out to be slightly smaller (Figure S7C). The average size of hES-derived mechanoreceptors [12], was larger than that determined for NOCL1-4 neurons, in agreement with smaller soma sizes found in native human nociceptors compared to LTMRs [34].

Of note, continuous expression of NGN1 (NOCL2 and NOCL4) led to smaller cell sizes compared to transient NGN1 expression (NOCL1 and NOCL3) (Figure S7C).

Extent and duration of after-hyperpolarizing currents are a defining feature of nociceptors and underlying ion channels of the HCN family. These channels have been implicated in pain signal transmission [49,50]. All four differentiation procedures (NOCL1-4) showed a prolonged rise time constant following the after-hyperpolarization potential (Figure 6B), similar to those reported for human and mouse nociceptors [12,48,51]. Interestingly, the average decay time of hES-derived LTMRs was shorter than that for those recorded in any of the four NOCL subpopulations. This correlates with HCN1 expression in the LTMRS (Figure 3C), a HCN variant largely absent from derived NOCL neurons and native mouse nociceptors [52]. Conversely, NOCL neurons, in particularly NOCL3s, expressed HCN2 (Figure 3C), an ion channel associated with pathological forms of pain [49,51].

Human and mouse nociceptors express both TTX (Tetrodotoxin)-sensitive and TTX-insensitive voltage-gated sodium (Na_V_-) channels. Both channel types have been implicated in pathological forms of pain in human patients and mouse models [35]. Accordingly, we found both types of conductances present in NOCL neurons. Irrespective of the differentiation protocol utilized, a small fraction (around 5–10%) of sodium currents was TTX resistant, similar to currents recorded in a subset of native murine small diameter DRG neurons that we sampled randomly (Figure S7D). Current–voltage relationships for NOCL3 in the presence of 300–500 nM TTX are indicative of a substantial contribution of Nav 1.8 currents, a marker for nociceptors (Figure S7E) [53]. Nav 1.8 contribution is further enhanced over time (≥24 days vs. ≥48 days in culture; Figure S7E inset).

In summary, characteristic ionic current properties of native nociceptors are recapitulated in derived NOCL neurons, with subtle variations observed across the 4 different differentiation procedures. Together, differences between NOCL1–4 neurons therefore resemble heterogeneity found in native DRG nociceptors.

### 3.6. Investigating the Role of PIEZO2 in hESC-Derived and Native Mouse Nociceptors

Many nociceptors are polymodal neurons that are able to respond to different stimuli such as temperature, acid, pungent chemicals and mechanical force. Other nociceptors are more specifically tuned to respond to discrete stimuli.

We found NOCL3 neurons to respond to noxious temperature as well as pungent substances such as capsaicin, while only a small fraction of NOCL1 and NOCL2 neurons responded to capsaicin (Figure 2B,C). This was also reflected in the higher TRPV1 expression in NOCL3 cells (Figure 3C).

**Figure 7:**
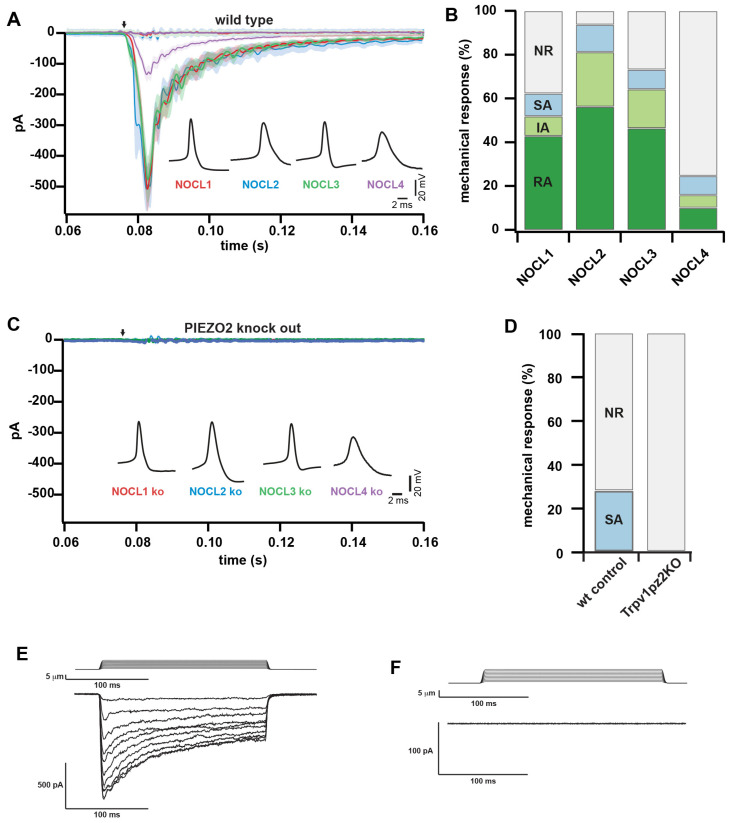
(**A**) Average currents (± SEM) of wild–type cells, evoked by a mechanical somatic displacement of 10 µm (arrow) are shown. Insets show example APs recorded from respective NOCLs. NOCL1 (*n* = 48 responders, *n* = 29 non–responders), NOCL2 (*n* = 38 responders, *n* = 11 non–responder), NOCL3 (*n* = 41 responders, *n* = 15 non–responders), NOCL4 (*n* = 17 responders, *n* = 52 non–responders). (**B**) Stacked bar charts show the percentage of neurons with rapidly adapting (RA), intermediate–adapting (IA) or slowly–adapting (SA) mechanical current as well as non–responders (NR). (**C**) Average currents (± SEM) of PIEZO2 KO neurons, evoked by a mechanical somatic displacement of 10 µm (arrow) are shown. Insets show example APs recorded from respective NOCLs. NOCL1 (*n* = 21), NOCL2 (*n* = 48), NOCL3 (*n* = 29), NOCL4 (*n* = 32). (**D**) Stacked bar charts show the percentage of mouse TRPV1/PIEZO2 wild–type or TRPV1 wild–type PIEZO2 knockout DRG neurons responding to mechanical stimulation with a slowly–adapting (SA) current or no current at all (NR). (**E**,**F**) currents of TRPV1/PIEZO2 wild–type (**E**) and TRPV1 wild–type PIEZO2 knockout DRG neurons evoked by increasing mechanical somatic displacement.

Next, we investigated whether derived nociceptors could respond to mechanical stimulation. Differentiated neurons were mechanically stimulated by indenting the somatic cell membrane using a nanomotor driven probe, while simultaneously recording somatic currents—a typical stimulation paradigm that recapitulates mechanosensitivity of native DRG sensory neurons [12,54]. Indentation of the cell membrane in steps of 1 µm resulted in increasing inward currents in many NOCL neurons, showing their ability to respond to mechanical stimulation (Figure 7A and Figure S7E).

Classically, native murine mechanosensitive currents are classified according to their inactivation kinetics into rapidly, intermediately and slowly inactivating currents [11]. While LTMRs typically inactivate rapidly, nociceptors are markedly heterogeneous and comprise cells displaying all three types of inactivation categories [55]. Indeed, we were able to record all three types of mechanical current categories in the three NOCL preparations (Figure 7B).

NOCL1, NOCL2 and NOCL3 neurons had comparable fractions of mechanosensitive neurons, while the NOCL4 procedure resulted in less neurons responding to membrane indentation (Figure S7E).

PIEZO2 has been identified as a primary mechanical transducer in non-nociceptive LTMRs [11,12]. However, both in murine and human nociceptors its contribution to mechano-nociception remains controversial [15,56].

We recently generated a hES cell line devoid of PIEZO2 [12]. Using this cell line, we first tested whether knockout hES cells were able to generate NOCL neurons. We found that their potential to differentiate was indistinguishable compared to that of wild-type control cells (Figure S8A,B).

We then tested whether PIEZO2 would contribute to mechanosensation. When comparing PIEZO2 knockout NOCL neurons with wild-type controls we found that no mechanically-inducible current remained, irrespective of the differentiation protocol used, while general electric excitability remained unaltered (Figure 7C and Figure S8C,D).

In agreement with the expression of PIEZO2 in native murine and human nociceptors [15,34,56,57,58] and with the expression of robust RNA of the mechanotransducer PIEZO2, all four different NOCL neuron types (Figure 3C and Figure 4C) resemble different subtypes of human nociceptors. These data suggest that PIEZO2 can act as a mechanotransducer in human-derived nociceptors when membrane intendation of cultured neurons is used as stimulation paradigm.

This highly penetrant phenotype was surprising, given that PIEZO2 appears to play little—if any—role in transducing painful mechanical stimuli in mice and humans [15,56]. We therefore wondered whether PIEZO2 is also required in native mouse nociceptors to mediate membrane indentation-induced mechanical responses, given that TRPV1 is a prototypical marker for nociceptive neurons that we found to be co-expressed with PIEZO2 in NOCL3 cells, we focused on TRPV1-expressing mouse nociceptors. To identify TRPV1-expressing sensory neurons, a Cre-dependent td-tomato expressing reporter virus was introduced into the dissociated sensory neuron cultures of Trpv1cre and Trpv1cre::Piezo2fl/fl animals. By 36 h after viral transduction, the td-tomato expressing neurons were analyzed electrophysiologically for the presence of mechanically induced currents. We found 27.8% of the TRPV1-expressing cells (5 out of 18) to respond to mechanical stimulation with a slow-adapting current (Figure 7D,E). This is in agreement with previous data, reporting Piezo2 being expressed in 24% of TRPV1 positive neurons [54]. In the absence of PIEZO2 in the TRPV1 positive neurons, none of the stimulated cells (*n* = 21) showed responses to mechanical stimuli (Figure 7D,F), supporting our finding, that PIEZO2 is indispensable for mechanical transmission in a specific subset of mechano-nociceptive sensory neurons in culture.

## 4. Discussion

Experiencing pain is an indispensable feature to protect bodily integrity. Lack of pain perception, as well as its transition to a chronic state on the contrary, can be devastating for the affected person. Investigating the changes in sensitization on a cellular and molecular level that could result in the switch of pain from an acute to a chronic state is therefore at the core of many research projects. We are exploring in how far the use of human pluripotent stem cell-derived nociceptor-like cells could provide a tool to complement rodent model systems that have been at the center of pain research for several decades. By using embryonic stem cell-derived neural crest precursors in combination with viral gene expression, we here describe the generation of human nociceptor-like cells. The molecular make-up and receptor armament of the generated sensory neurons varied as a function of (i) the duration of *NEUROGENIN1* expression and (ii) the availability of serum and/or neurotrophic factors in the culture medium.

One striking feature of the system is the induction of a differentiation program that —when combined with a positive selection procedure—allowed the production of a homogenous population of sensory neurons that display functional hallmarks of prototypical nociceptors. One protocol in particular resulted in the generation of a homogenous population of TRPV1-expressing nociceptor-like cells, as was assessed by functional Ca imaging as well as in situ hybridization experiments. Additionally, the cells showed the ability to become sensitized by inflammatory mediators such as NGF. Furthermore, the derived nociceptor-like cells responded to temperature stimulation, another characteristic feature of TRPV1-expressing nociceptors [9]. In comparison to cultured mouse DRG neurons or several other differentiation protocols [3,4,5], the homogenous response of the human ESC-derived neurons to capsaicin is its most striking asset, showing the potential of the cellular system as a model for studying specific sensory neuron subpopulations. The added benefits described here are the result of a two-step differentiation protocol that first generates neural crest-like cells, the natural precursors of sensory neurons, and secondly differentiates them further into sensory neurons. Recapitulating this developmental intermediate (neural crest cells) promotes more efficient sensory neuron generation, compared to other protocols that attempt to transdifferentiate fibroblasts or hES/iPS cells into nociceptors directly, and thereby generate either mixed populations of sensory neurons or rather low amounts of specific subtypes together with non-neuronal cells [4,5]. Another advantage of the described differentiation approach is the high level of reproducibility ensured by the induced expression of the NGN1 transcription factor compared, for example, to the use of exogenous factors alone [3].

Previous cellular classifications, largely based on work in rodents, have used marker gene expression profiles to categorize subgroups of nociceptors [1]. The advent of single-cell RNAseq methods has broadened this type of molecular classification and has highlighted ever smaller and more refined subgroups of sensory neurons [31,32,59,60,61]. An observation in agreement with recent single-cell sequencing data derived from primate DRGs (macaques, humans, [25,26,62]). Nonetheless, molecular differences between human and rodent sensory neurons have been observed, such as the more widespread overlap of the two neurotrophic receptors *TRKA* and *RET* in mature human nociceptors [34,39], a feature that we also find in the derived NOCL neurons. Notwithstanding, one limitation of our approach is that it is difficult to unambiguously determine what precise *native* nociceptor subtype the respective NOCL neurons correspond to. Despite the increasing amount of primate single-cell data derived from DRGs [25,26,62], the datasets are still limited in numbers of sequenced cells/nuclei; therefore, the depth of the derived reads which might explain the differences between these studies in defining the sensory neuron subtypes present in primate DRGs. Additionally, Sharma et al., 2019 [61] have shown that the sensory subtype composition of mouse DRGs varies across axial levels, which can have an impact on the results if only DRGs from distinct levels are used for sequencing experiments. Deconvoluting cell type proportions in our sequenced samples using data either from Tavares-Ferreira et al., 2022 [26] or Nguyen et al., 2021 [25] shows that our cells, albeit their homogenous appearance regarding capsaicin responsiveness for example, do not fall into only one category of sensory subtype but several, based on combined marker analysis. This could mean two things: (1) our cells, after a culture period of 34 days, comprise a rather heterogenous population of nociceptive subtypes, most of them expressing functional TRPV1 or (2) our cells are not fully mature and stuck at some developmental state. This is not surprising given that our in vitro differentiation procedure does not fully recapitulate in vivo sensory neuron differentiation, which involves temporally and spatially refined cues as well as target organ innervation. Additionally, recent data obtained in mice showed that developing sensory neurons undergo mixed lineage stages before acquiring their final identity [60]. Present work in our lab is focusing on additional ways to push the cells into more mature states and thereby also into more well defined and separated sensory subtypes

Nevertheless, when we assessed cell type composition of the derived NOCL neurons using previously generated human molecular nociceptor profiles [26], we found NOCL1 cells to share most similarities with PENK^+^ or TRPA1^+^ nociceptors besides others. NOCL2 cells on the other side share markers mostly with cold nociceptors and Aδ-HTMRs while the majority of NOCL3 cells resembles Aδ-HTMRs or pruritogen receptor enriched neurons. These results clearly show that the changes in NEUROGENIN expression (10 days versus constantly expressed) do influence the generation of the derived sensory neuron subtypes, as well as the addition of exogenous factors such as serum.

The heterogeneity of endogenous nociceptors is also reflected in their functional properties, such that certain subgroups of sensory neurons are tuned to detect heat, cold, chemical and/or mechanical stimuli [63]. Particularly important pathologically (and difficult to treat therapeutically) are mechano-nociceptive characteristics: the ability to respond to noxious (and thus painful) mechanical forces. We investigated the mechanotransduction properties of our derived nociceptor populations and found most of them to be responsive to mechanical stimulation.

A major unresolved question pertains to the molecular mechanism(s) that enable nociceptive neurons to detect and respond to painful mechanical stimuli. One protein, PIEZO2, has recently gained prominent attention because it was found to be the main transducer of innocuous mechanical stimuli (such as touch) in non-nociceptive LTMRs, both in mice and humans [11,12,13]. Concurrently, the gene was also found to be expressed in a wide range of human and mouse nociceptors (of both, peptidergic and non-peptidergic origin), raising the eminent possibility that the receptor is also required for mechano-nociception. In vitro experiments by Dubin et al. [64] using cultured mouse DRG neurons showed an increase in PIEZO2-dependent current amplitude and a slowing of its inactivation upon stimulating inflammatory pathways, suggestive of a role for PIEZO2 in inflammatory pain. Another study demonstrated a connection between capsaicin-induced TRPV1 activation and subsequent PIEZO2 inhibition in nociceptors based on the depletion of membrane phosphoinositides [65]. Additionally, in *d. melanogaster,* the PIEZO2 analog *dpiezo* is required for mechano-nociception but not for innocuous gentle touch [66]. While these previous studies argue in favor of a role for PIEZO2 in mechano-nociception, more recent in vivo studies suggest that PIEZO may only play a minor—if any—role for this highly medically relevant property. One study, genetically deleting PIEZO2 in selective neurons in mice, suggested that the mechanotransducer plays a minor role in detecting painful stimuli such as pinch or pinprick [56]. In another study, focusing on 4 human patients with point mutations in the *PIEZO2* gene, a contribution for a function of the protein in acute mechano-nociception could not be found [15].

To our surprise and in contrast to what has been reported from the aforementioned patient studies, the absence of PIEZO2 in the hESC-derived nociceptor-like cells, while we were using a previously generated PIEZO2 knockout hESC line [12], showed the loss of mechanotransduction in all generated subpopulations. We found a similar result in cultured mouse nociceptors, where about 27% of TRPV1-expressing mouse neurons showed mechanotransduction properties, while none could be detected in the absence of PIEZO2.

The discrepancy between the patient studies and our results could be partly due to the mechanical stimulation paradigm used in the cell culture to mimic physiological mechanical pain stimuli: high-frequency indentation of the plasma membrane using a nanomotor-driven probe. While our results suggest that there is a requirement for PIEZO2 in indentation-mediated mechanotransduction at the cell soma in both types reviewed, human ESC-derived nociceptor-like cells as well as in a subset of primary mouse TRPV1-expressing DRG neurons, additional mechanisms may contribute to mechanosensitivity under in vivo conditions. This took place for example in cutaneous Schwann cells, as has recently be shown by Abdo et al., 2019 [67].

Another possible explanation could be a potential contribution of other mechanosensitive ion channels in nociceptors of patients devoid of functional PIEZO2. Several other ion channels with mechanosensitive properties have been described in DRG neurons [68] and their mechanosensitive properties may become unmasked to play more prominent roles in the absence of PIEZO2.

In summary, our differentiation protocols allow for the generation of several different nociceptor subtypes that bear prominent molecular and functional similarity to human and rodent nociceptor subgroups. Therefore, it offers the possibility to study aspects of sensitization on a cellular and molecular level in a human genetic background and thereby can serve as a tool to complement data derived from rodent model organisms. The versatility of the differentiation procedures (usable for embryonic and patient-derived pluripotent stem cells), the effective generation of homogenous populations of functional polymodal nociceptor-like neurons and the ability for CRISPR gene editing make these cells an important addition in pain research.

## Data Availability

RNAseq data derived from the generated cells were deposited on ArrayExpress under the following accession numbers: E-MTAB-12098 and E-MTAB-12099. Single-cell RNAseq data from Nguyen et al., 2021 [25] were downloaded from GEOBUS (GSE168243) and the associated cell labels were provided by the authors upon request. Spatial transcriptomic data from Tavares-Ferreira et al., 2022 [26], and the spot identities were downloaded from a publicly available platform provided by the authors (https://sensoryomics.shinyapps.io/RNA-Data/, 15 August 2022).

## Supplementary Materials

The following supporting information can be downloaded at: https://www.mdpi.com/article/10.3390/cells11182905/s1, Figure S1: Generation of hESC-derived nociceptor-like neurons; Figure S2: Generation of *TRKA*-tomato hESC reporter cell line; Figure S3: Calcium imaging experiments of various differentiated cells; Figure S4: Time-course of selected genes in NOCL3 neurons; Figure S5: Molecular characterization of NOCL1 and NOCL3 neurons; Figure S6: Classification of hESC-derived sensory neuron subtypes; Figure S7: Electrophysiological characterization of the derived cells; Figure S8: Molecular and electrophysiological characterization of PIEZO2 KO NOCL neurons.
