# Supplementary material for: Human Stem Cell-Derived TRPV1-Positive Sensory Neurons: A New Tool to Study Mechanisms of Sensitization
Source: Cells. 2022 Sep 17;11(18):2905. doi: 10.3390/cells11182905 (PMC9497105; doi:10.3390/cells11182905)
Supplement: Supplementary file 1 [file cells-11-02905-s001.zip › cells-1892327-supplementary.pdf]

Supplementary Figure S1 A

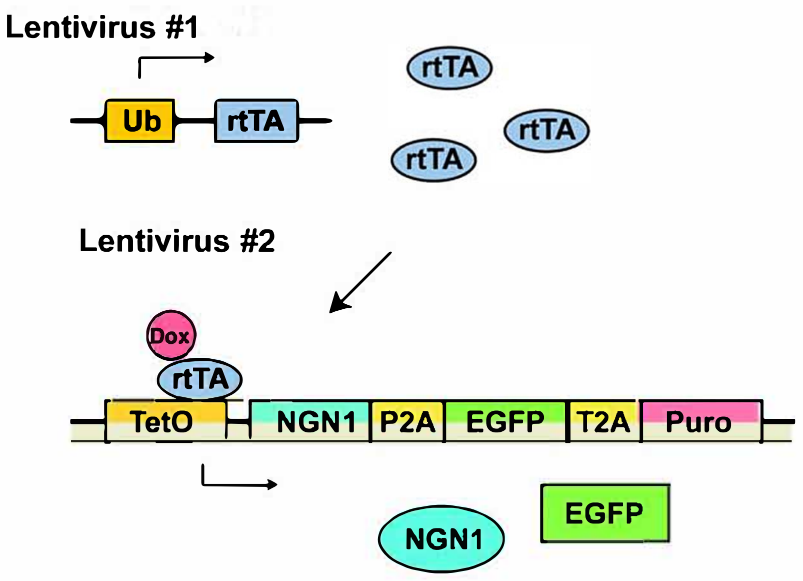

B

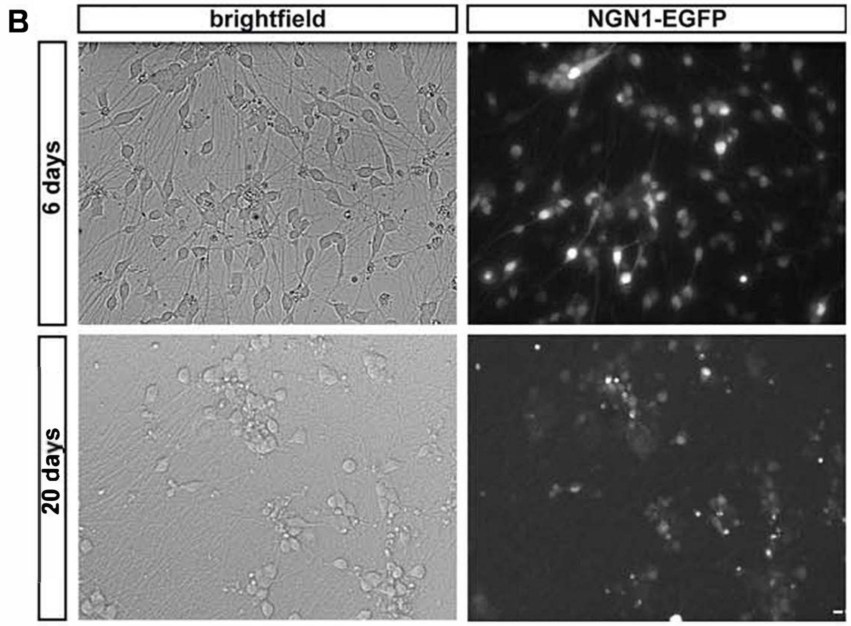

C

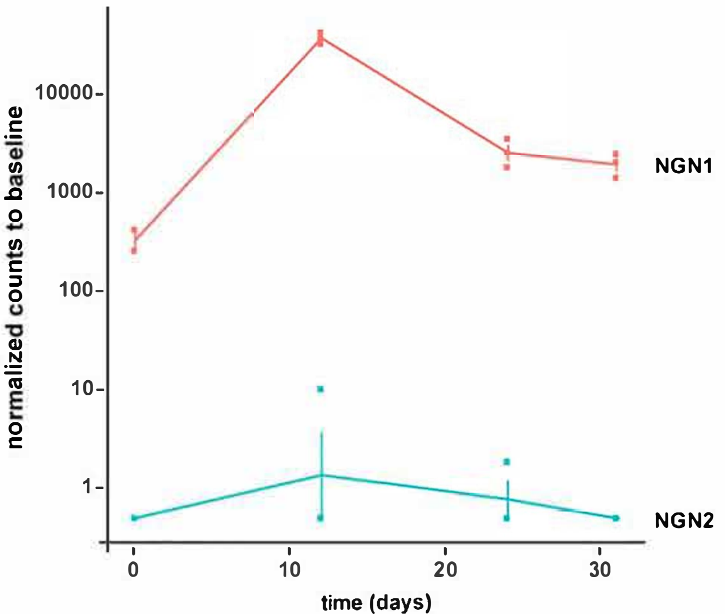

Supplementary Figure S1

Generation of hESC-derived nociceptor-like neurons

(A) Schematic drawing of the two lentiviruses used for infection of hESC-derived NCLCs. Lentivirus #1 expresses the transactivator rtTA driven by the ubiquitin- (Ub) promoter. Lentivirus #2 contains the tetracycline promoter site (tetO), were the transactivator can bind and induce –in the presence of doxycline (Dox)– the expression of *NEUROGENIN1* (*NGN1*), enhanced green fluorescence protein (EGFP) and the puromycin resistance cassette (Puro), all of which are linked via P2A and T2A sequences.

(B) Live cell imaging showing the expression of EGFP in infected hNCLCs after 6 and 20 days in culture. Cells were exposed to doxycycline-containing medium for 10 days. Strong EGFP signal is observed after 6 days while the signal is almost absent after 20 days. Scale bar 20µm.

(C) Time course of *NEUROGENIN1* (*NGN1*) and *NEUROGENIN2* (*NGN2*) transcript-levels after 0, 12, 24 and 31 days of NOCL3 differentiation. Data are shown as normalized counts ± SEM. N=3.

Supplementary Figure S2

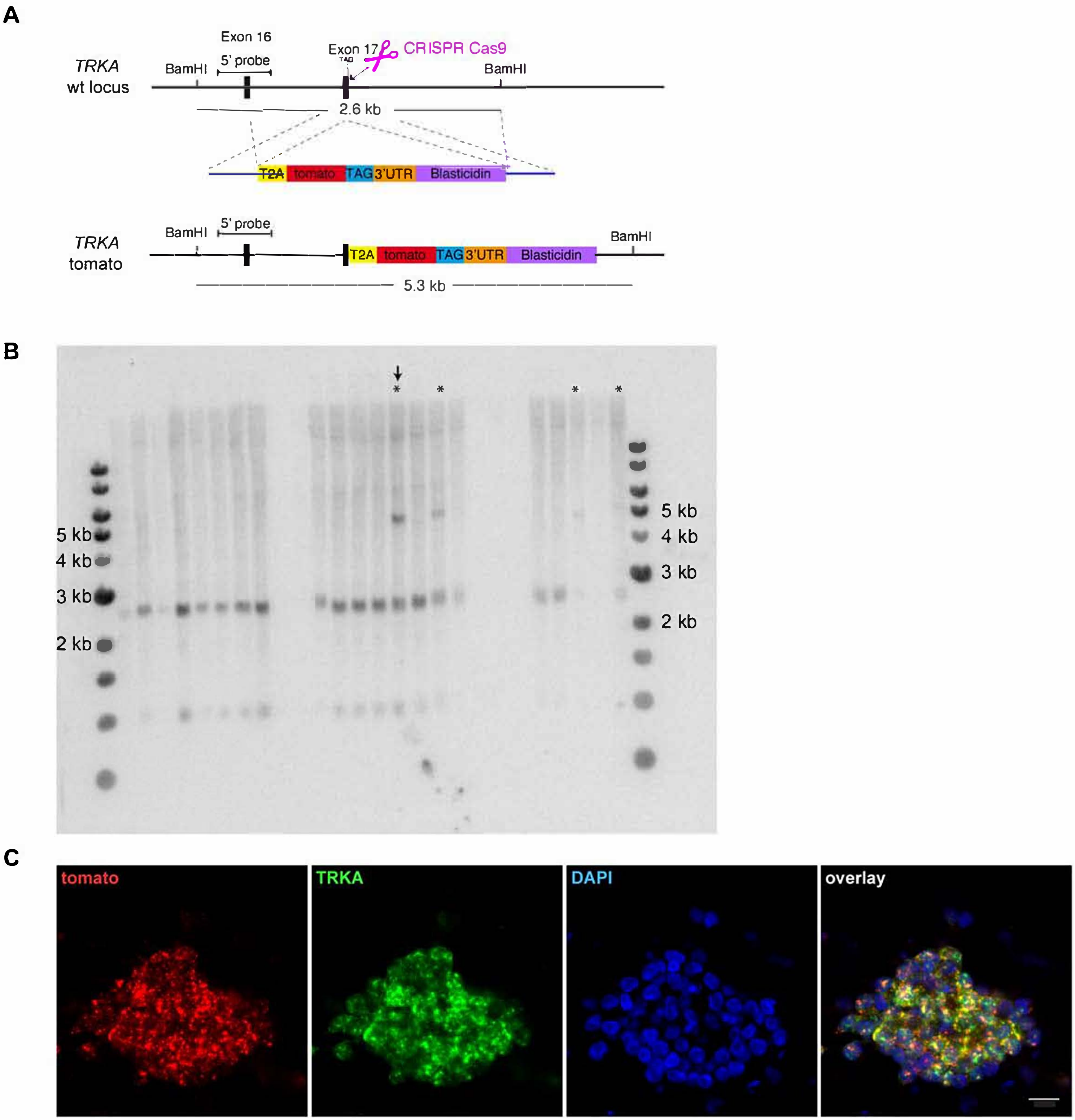

Supplementary Figure S2

Generation of *TRKA*-tomato hESC reporter cell line

(A) Schematic illustration indicating the cloning strategy used to generate a hESC-*TRKA*-tomato reporter line. The tandem tomato sequence was introduced into the last exon (exon 17) of the *TRKA* locus before the stop codon (TAG) via a T2A linker sequence using CRISPR/Cas9 technology. Additionally, a blasticidin selection cassette was inserted in the *TRKA* 3'UTR area with the same cloning vector. To identify hESC clones with the correct genetic modification, a 5' southern probe was designed recognizing a sequence around exon 16.

(B) Southern blot done with DNA from 26 hESC clones, showing the presence of 4 positive clones indicated with an asterisk. Positive clones contain the wild type band at 2.6 kb as well as a band at 5.3 kb, indicating the correct incorporation of the reporter-sequence. The clone marked with an additional arrow was used for most differentiation procedures.

(C) *In situ* hybridizations for tomato (red) and *TRKA* (green) expression in NOCL1 cells demonstrate co-expression of both markers in 98.7 ± 0.9% of cells. Nuclear marker DAPI in blue; scale bar 20 µm; N=3.

Supplementary Figure S3

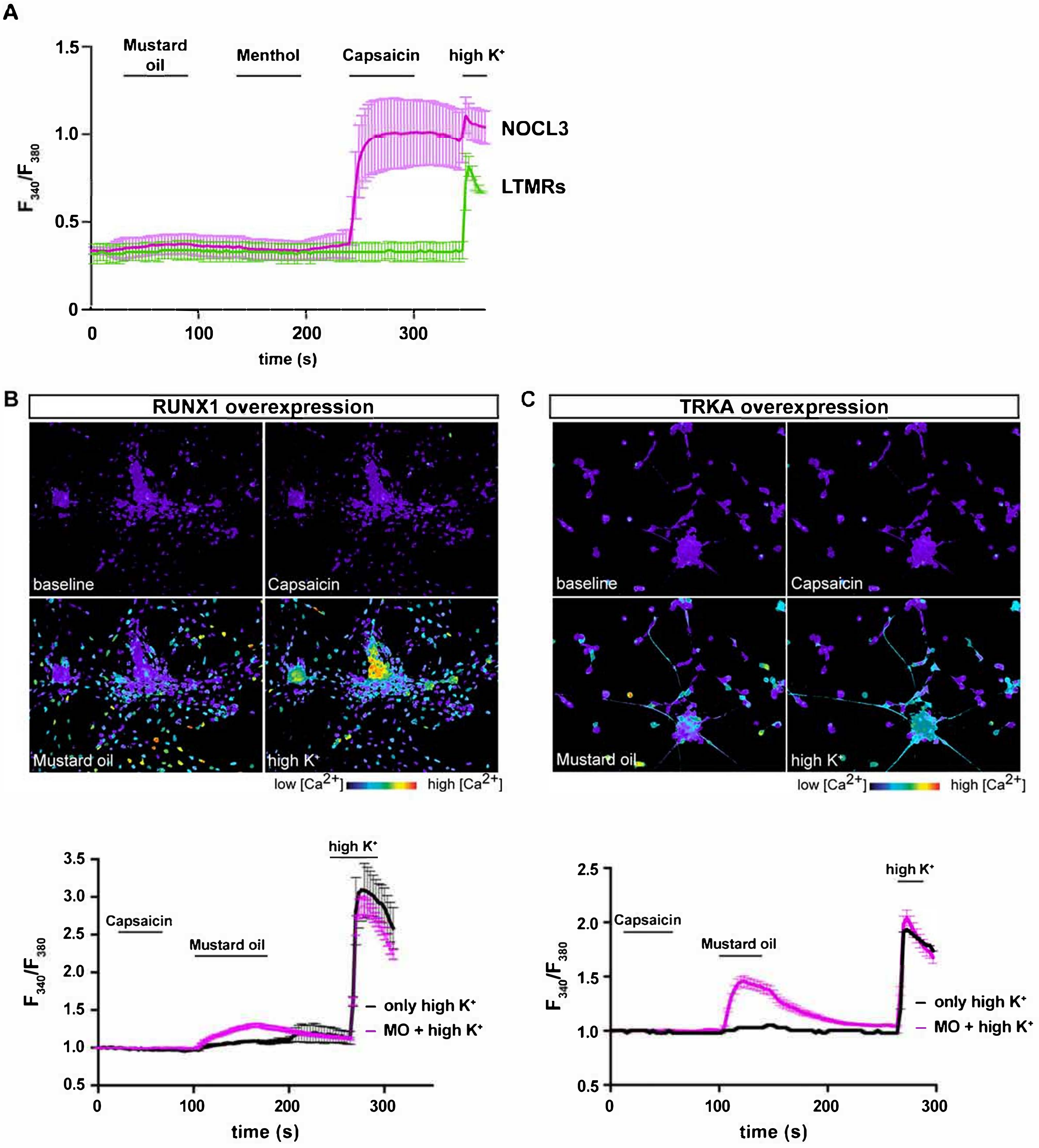

Supplementary Figure S3

Calcium imaging experiments of various differentiated cells

(A) Representative fluorescence ratios (340nm/380nm) of hESC-derived low threshold mechanoreceptors (LTMRs, green) and NOCL3 cells (pink) loaded with the calcium indicator Fura2 after challenging them with mustard oil (200µM), menthol (500µM) or capsaicin (1µM). To visualize all neurons a stimulus of high K^+ Ringer solution was added at the end. LTMRs did not respond to any stimulus besides high K^+ Ringer solution. Data are shown as average ratio ± stdev (NOCL3 = 123 cells; LTMRs = 2 cells).

(B) Representative pseudo-colour images of calcium responses in hESC-derived NCLCs differentiated after infection with a RUNX1 expressing lentivirus, before (baseline) and during capsaicin or mustard oil stimulation. High K^+ Ringer solution was added at the end. Lower panel shows representative normalized fluorescence ratios (340nm/380nm) of the same cells, indicating absence of any capsaicin responders but a small fraction of mustard oil responders under these conditions.

(C) Representative pseudo-colour images of calcium responses in hESC-derived NCLCs differentiated after infection with a *TRKA* expressing lentivirus, before (baseline) and during capsaicin or mustard oil stimulation. High K^+ Ringer solution was added at the end. Lower panel shows representative normalized fluorescence ratios (340nm/380nm) of the same cells, indicating absence of any capsaicin responders and presence of a small fraction of mustard oil responders under these conditions.

Supplementary Figure S4

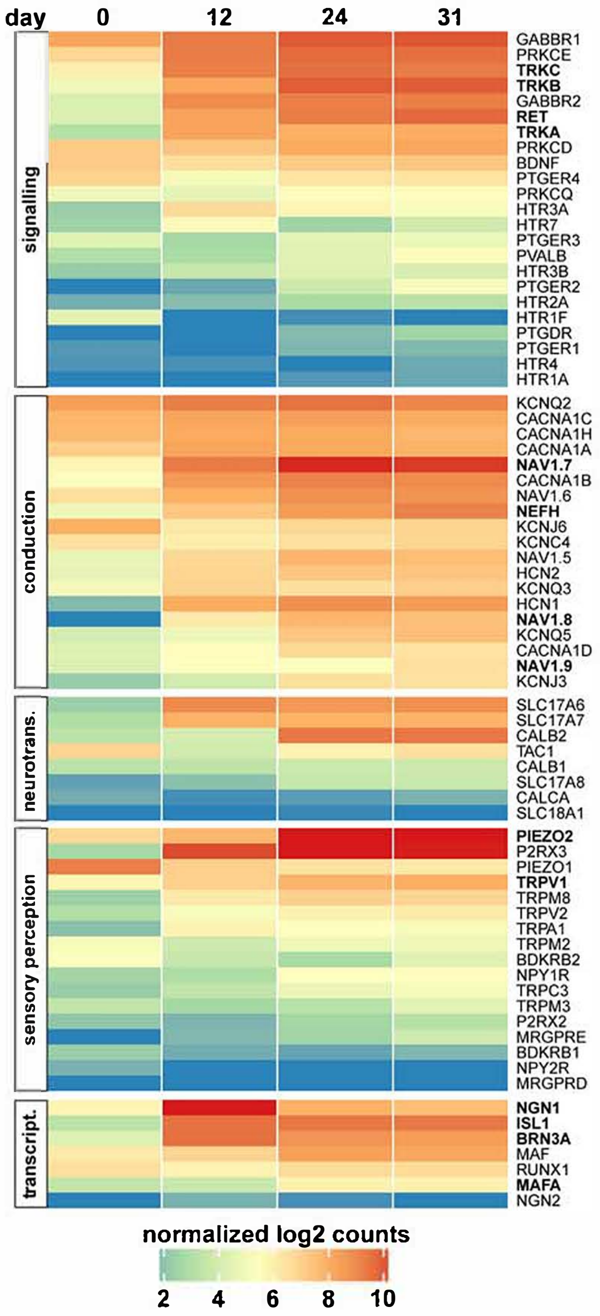

Supplementary Figure S4

Time-course of selected genes in NOCL3 neurons

Heat map showing expression (normalized log2 counts) of gene transcripts during the course of NOCL3 development (samples were taken at 0, 12, 24 and 31 days, N = 3 per time point). Genes are categorized into functionally connected groups and those indicated bold have been analysed in more detail (neurotrans. = neurotransmission, transcript. = transcription).

Supplementary Figure S5

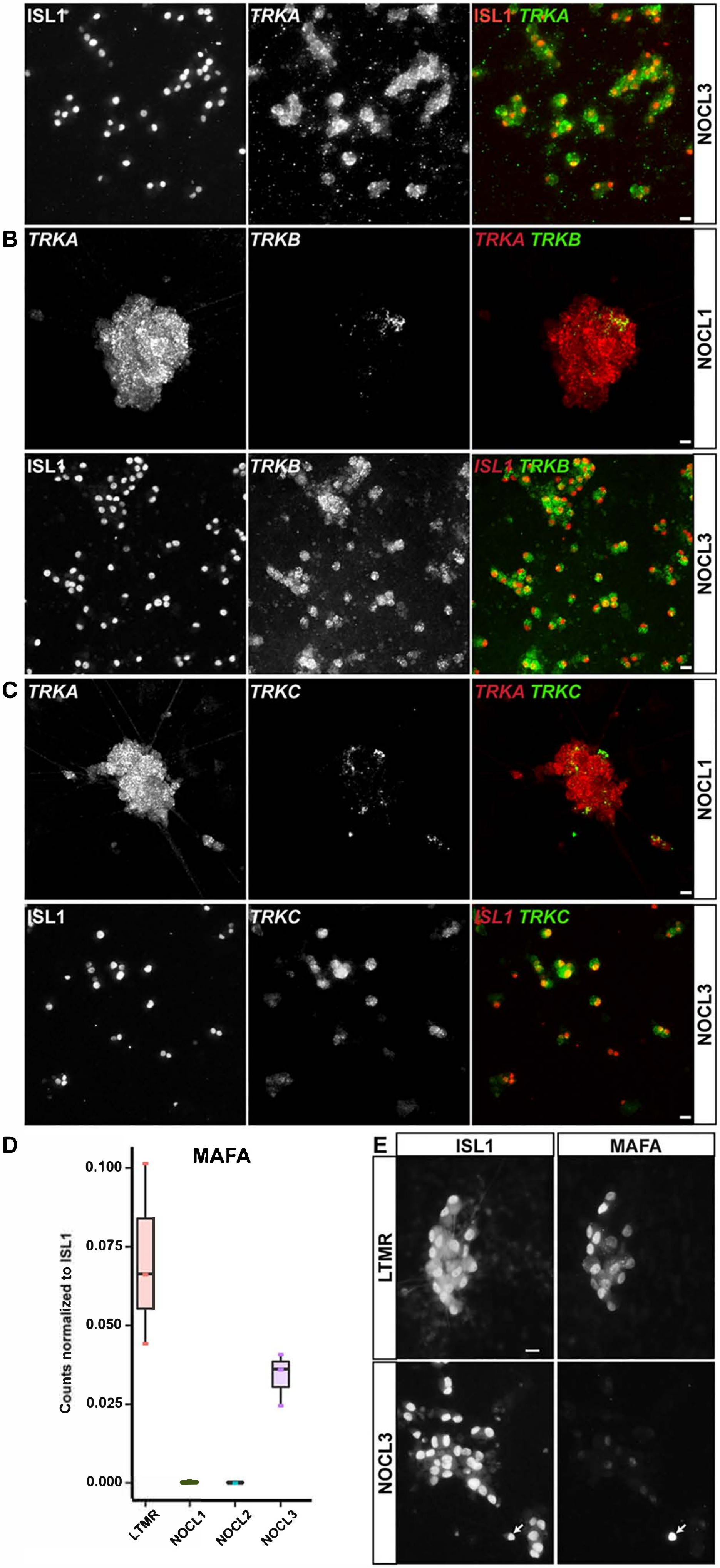

Supplementary Figure S5

Molecular characterization of NOCL1 and NOCL3 neurons

(A) Immunostaining and *in situ* hybridization of NOCL3 neurons with anti-ISL1 antibody (left and red) and a *TRKA*-probe (middle and green), showing that essentially all sensory neurons express *TRKA* (99.7 ± 0.3%; N=3).

(B) *In situ* hybridization of NOCL1 neurons (upper panel) for *TRKA* (left and red) and *TRKB* (middle and green) and immunostaining plus *in situ* hybridization of NOCL3 neurons (lower panel) using an anti-ISL1 antibody (left and red) and a *TRKB* probe (middle and green) to show the presence of *TRKB*-transcripts in different NOCL cells. While NOCL1 neurons only rarely show presence of *TRKB*, the majority of NOCL3 neurons do express it.

(C) Dual-color *In situ* hybridization of NOCL1 neurons (upper panel) for *TRKA* (left and red) and *TRKC* (middle and green) and immunostaining and *in situ* hybridization of NOCL3 neurons (lower panel) using an anti-ISL1 antibody (left and red) and a *TRKC* probe (middle and green) to show the presence of *TRKC* transcripts in different NOCL cells. The fraction of NOCL1 neurons expressing *TRKC* transcripts is minor, compared to NOCL3 neurons.

(D) Comparison of MAFA transcript-levels between LTMRs and NOCL1–3 neurons, indicating that the NOCL3-differentiation procedure results in slightly higher MAFA transcript-levels compared to those of NOCL1 or NOCL2-differentiation procedures. LTMRs exhibit the highest MAFA levels (RNAseq MAFA transcript counts were normalized to *ISL1* transcript counts (N=3)).

(E) Immunostaining of hESC-derived LTMRs and NOCL3 neurons using an anti-ISL1 antibody (left panel) and an anti-MAFA antibody (right panel), showing that the level of MAFA-protein in NOCL3 neurons is minor in comparison to LTMRs, despite the presence of some MAFA transcript in NOCL3 neurons (**d**) (arrow indicates a MAFA-positive NOCL3 cell). Scale bars in all figures: 20µM.

Supplementary Figure S6

A comparison to Tavares-Ferreira et al., 2022

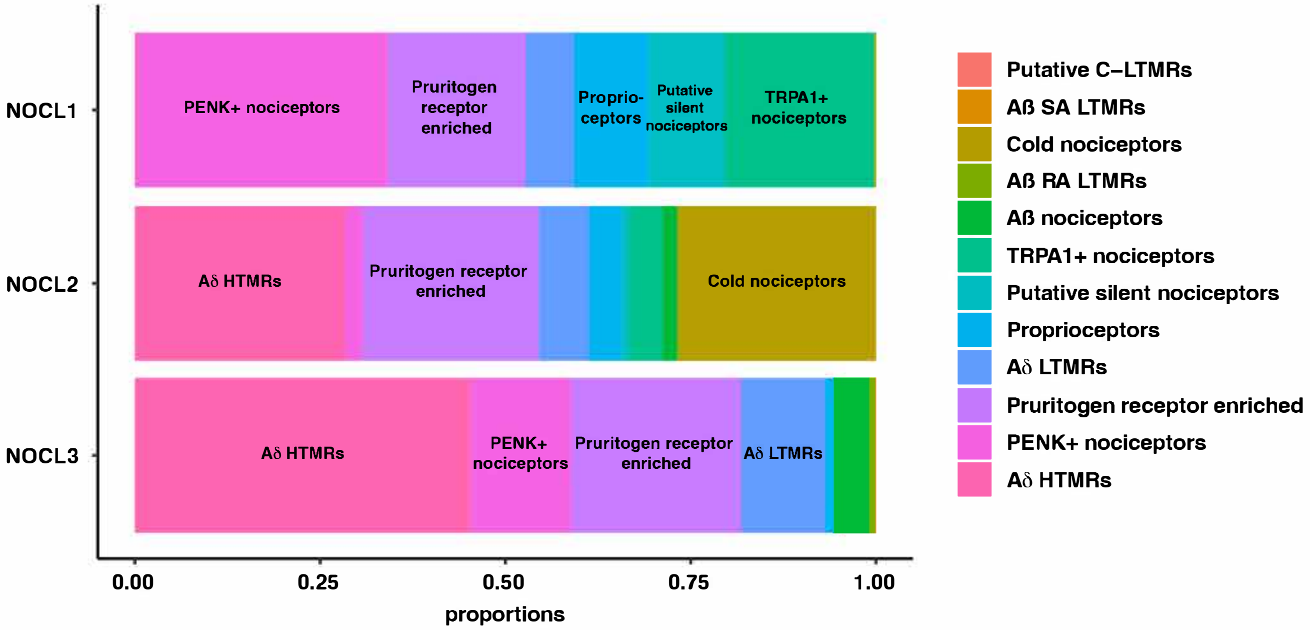

B comparison to Nguyen et al., 2021

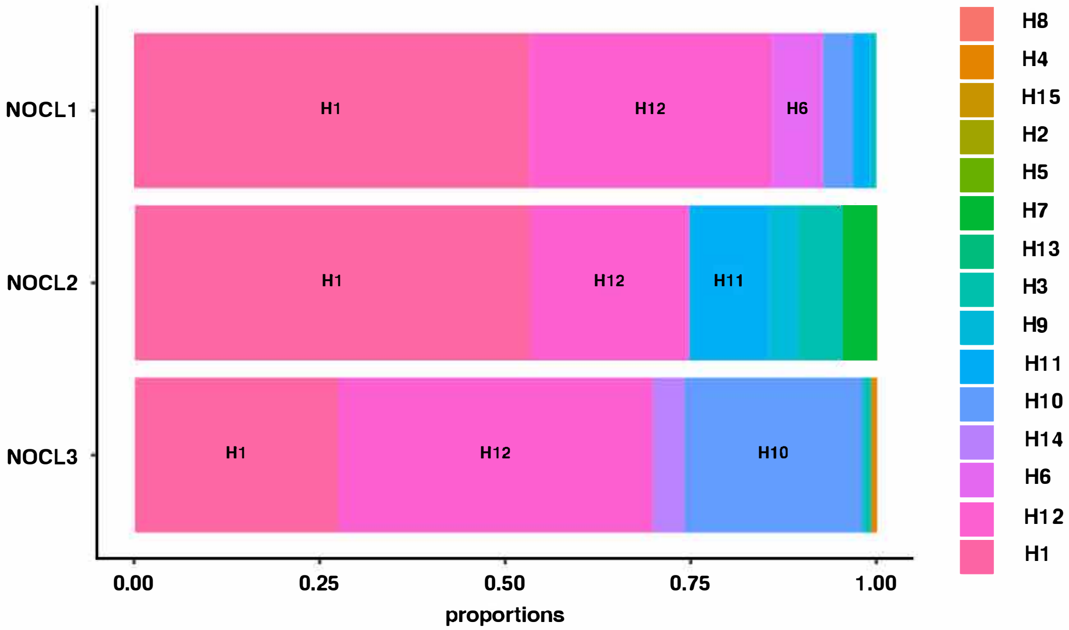

C comparison to Tavares-Ferreira et al., 2022

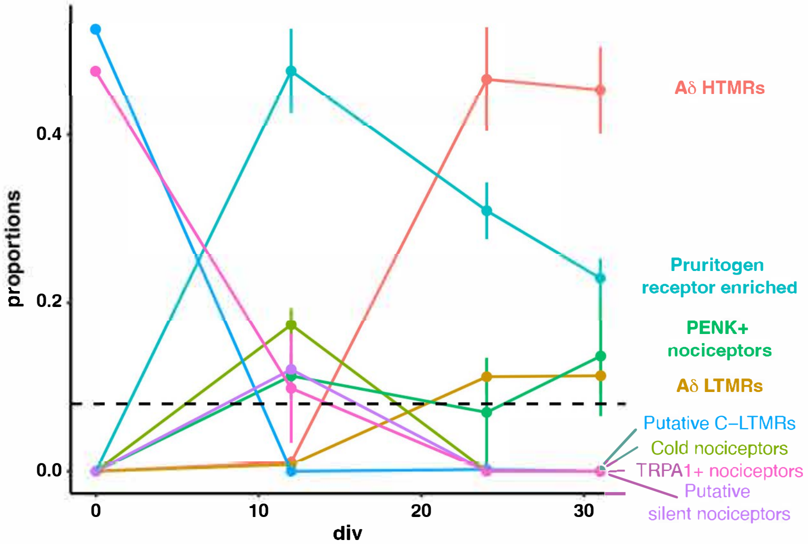

Supplementary Figure S6

Classification of hESC-derived sensory neuron subtypes

(A) and (B) show bar plots of deconvoluted cell-type proportions from NOCL1-3 sequencing data using published spatial and single-cell sequencing datasets of human DRG neurons from Tavares-Ferreira et al., 2022 (A) or Nguyen et al., 2021 (B), respectively. (C) Deconvoluted cell-type proportions of NOCL3 sequencing data derived over the course of development, assessed using the dataset from Tavares-Ferreira et al., 2022.

supplementary Figure S7

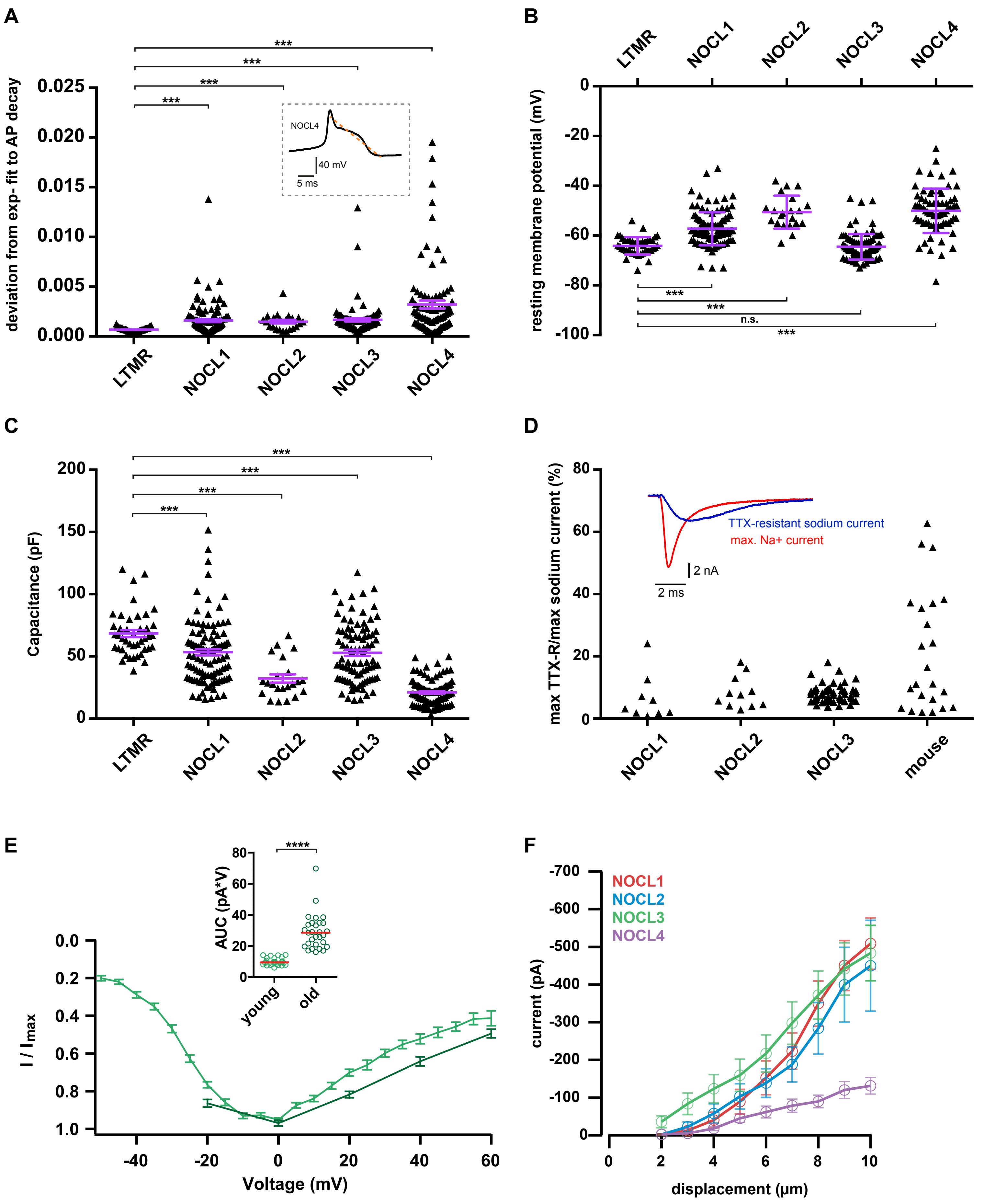

Supplementary Figure S7

Electrophysiological characterization of the derived cells

(A) Analysis of action potential (AP) waveforms. The AP repolarization was analyzed as the deviation from a single exponential fit between the AP peak and the peak of the afterhyperpolarization potential as depicted in the inset and compared to the result obtained for derived LTMRs; LTMR (n=43), NOCL1 (n=109), NOCL2 (n=70), NOCL3 (n=90) and NOCL4 (n=87), *** ($p \leq 0.0002$), Mann-Whitney test. (B) Resting membrane potential V_m was measured in current clamp for LTMR (n=44), NOCL1 (n=108), NOCL2 (n=70), NOCL3 (n=90) and NOCL4 (n=71). n.s. ($p \geq 0.1432$), *** ($p < 0.0001$), Mann-Whitney test. (C) Capacitance was measured as an estimation of cell size for LTMR (n= 42), NOCL1 (n=109), NOCL2 (n=70), NOCL3 (n=90) and NOCL4 (n= 99), *** ($p \leq 0.0002$), Mann-Whitney test. (D) Percentage of the ratio of the maximal TTX-resistant sodium current and the maximal sodium current, measured in current-voltage curves respectively. (A-D) In all graphs: each triangle in a scatter plot corresponds to one measured cell and mean ± SEM is depicted. E) Normalized current-voltage curves (means ± SEM) for NOCL3 ≥ 24 days in culture (green, n=26) and ≥ 48 days in culture (dark green, n=29) in the presence of 300-500 nM TTX to isolate TTX-R currents. Inset: Area under the curve (AUC) ranging from -20 mV to 60 mV in 20 mV steps, **** ($p < 0.0001$), red lines are the median. (F) Average (± SEM) current of neurons responding to a mechanical stimulation evoked by increasing displacement of the somatic cell membrane.

Supplementary Figure S8

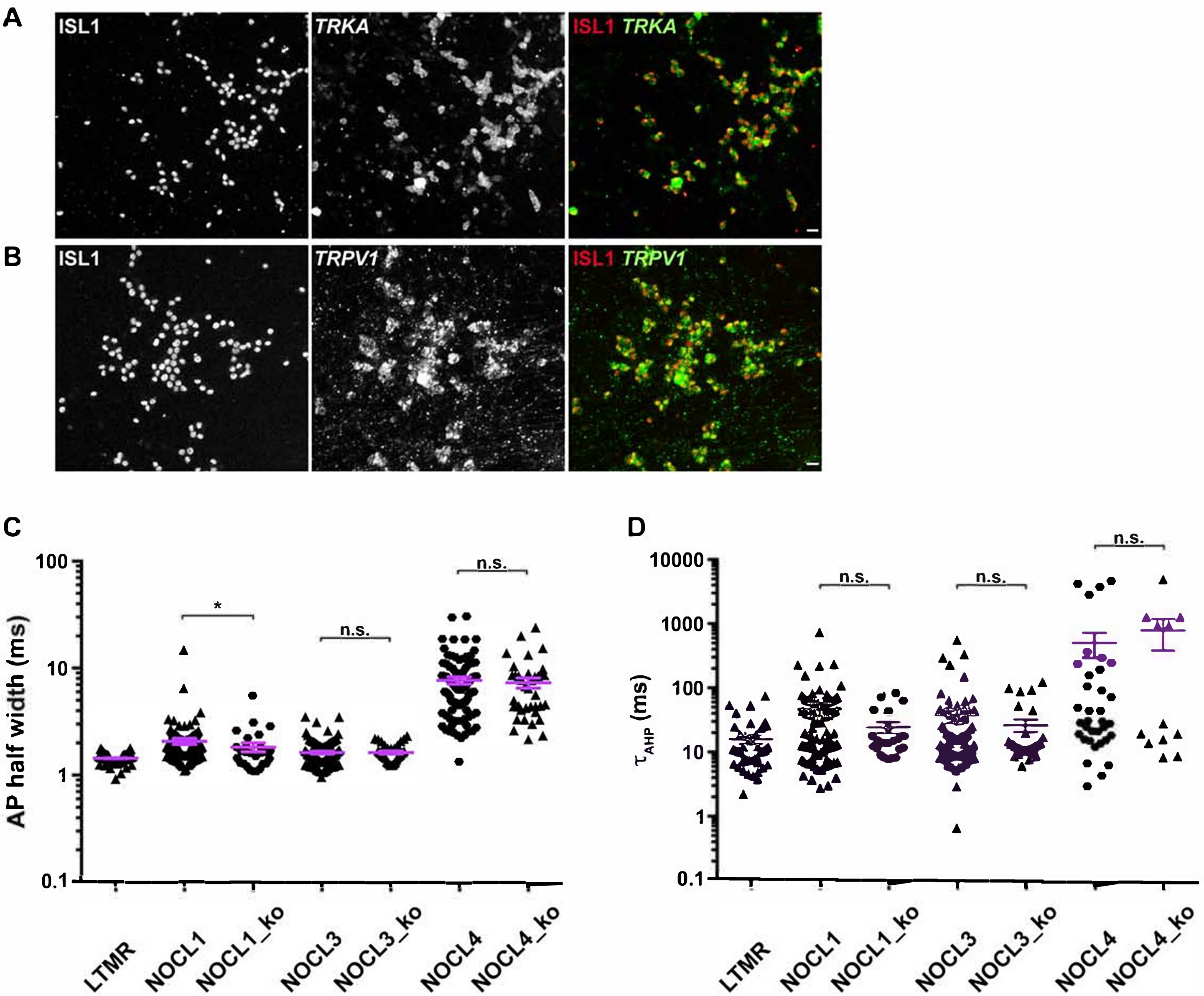

Supplementary Figure S8

Molecular and electrophysiological characterization of PIEZO2 KO NOCL neurons

(A) Immunostaining and *in situ* hybridization of hESC-derived PIEZO2 knockout NOCL3 cells with an anti-ISL1 antibody (left and red) and a *TRKA*-probe (middle and green), indicating that almost all ISL1-positive cells are also *TRKA*-positive.

(B) Immunostaining and *in situ* hybridization of hESC-derived PIEZO2 knockout NOCL3 cells with an anti-ISL1 antibody (left and red) and a *TRPV1*-probe (middle and green), indicating that almost all ISL1-positive cells are also *TRPV1*-positive. Scale bars 20 µm.

(C) Somatic AP half width is similar between wild type and *PIEZO2* KO neurons, as assessed by Mann-Whitney test, * ($p=0.0261$), n.s. ($p \geq 0.0581$). (D) Decay of the after hypolarization potential (AHP) τ_{AHP} is similar between wild type and *PIEZO2* KO neurons as assessed by Mann-Whitney test, n.s. ($p \geq 0.6226$). In graphs **c**) and **d**), each marker in the scatter plot corresponds to one measured cell and mean ± SEM is depicted.
